# Supplementary material for: Non-canonical pathway for Rb inactivation and external signaling coordinate cell-cycle entry without CDK4/6 activity
Source: Nat Commun. 2023 Nov 29;14:7847. doi: 10.1038/s41467-023-43716-y (PMC10687137; doi:10.1038/s41467-023-43716-y)
Supplement: Supplementary file 1 — Supplementary Information [file 41467_2023_43716_MOESM1_ESM.pdf]

Supplementary Information

Non-canonical pathway for Rb inactivation and external signaling sequentially coordinate cell-cycle entry without CDK4/6 activity

Supplementary Figures. 1–11.

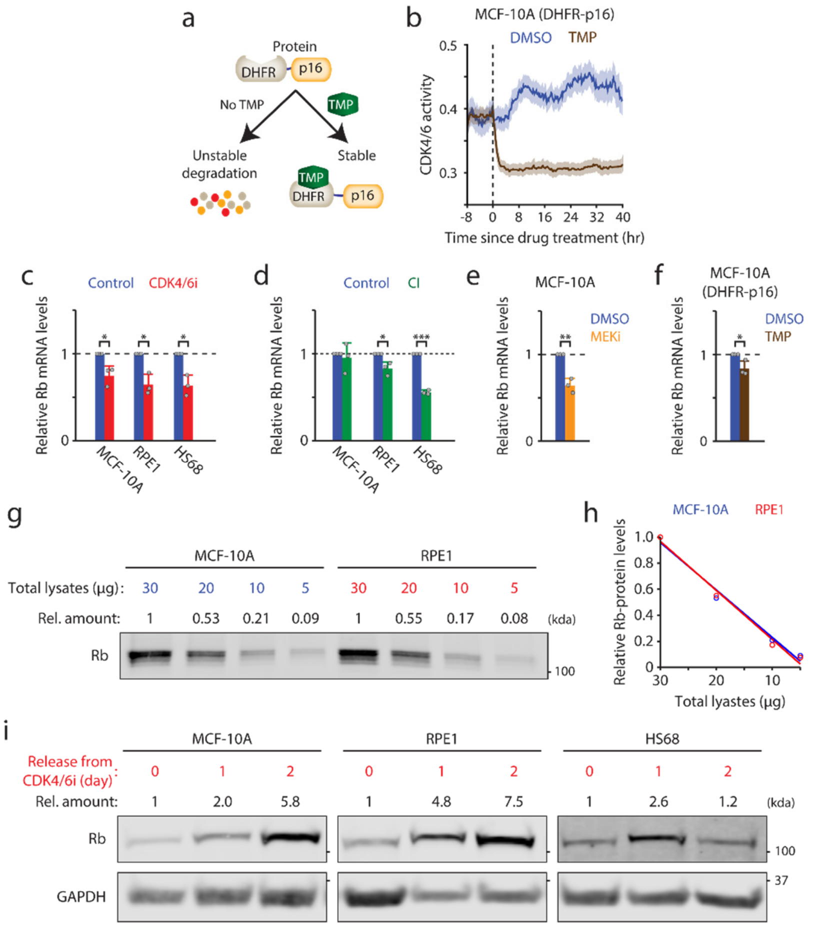

Supplementary Figure 1. Expression levels of Rb following CDK4/6 inhibition.

a Schematic illustrating the DHFR-TMP protein stabilization system used to induce exogenous p16 expression. **b** Average traces of CDK4/6 activity in MCF-10A cells with stable DHFR-p16 construct expression after treatment with either DMSO or TMP (50 μM). The black dotted line indicates the drug-treatment time. Data are shown as mean ± 95% CI (DMSO: n = 551 cells; TMP: n = 1,492 cells). **c**–**f** Relative Rb mRNA levels after palbociclib (1 μM) treatment (**c**), contact inhibition (**d**), trametinib (10 nM) treatment (**e**), or p16 overexpression induced by TMP (50 μM) treatment (**f**) for 48 hr. Data are shown as mean ± SD (n = 3 biological replicates). Asterisks indicate significant differences in the two-tailed unpaired t-test (*$p \leq 0.05$; **$p \leq 0.001$; ***$p \leq 0.0001$). **g** Immunoblot showing Rb levels across various amounts of total lysates in MCF-10A and RPE1 cells. **h** Relative Rb-protein levels. Solid lines represent the best fitted lines. **i** Immunoblot showing Rb and GAPDH expression in cells released from 48 hr-treatment with palbociclib (1 μM). Relative amounts indicate Rb-band intensities after normalizing with GAPDH.

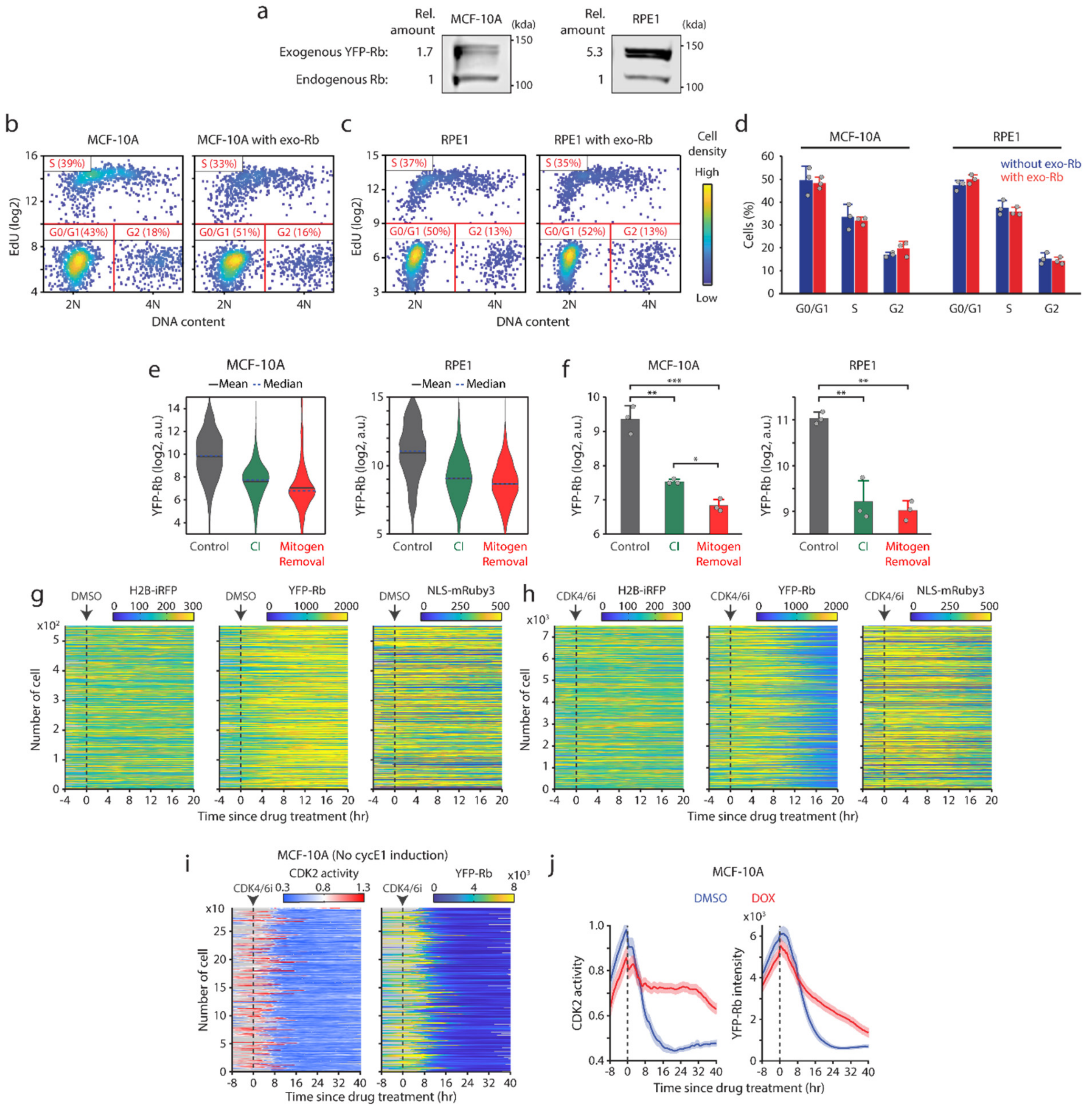

Supplementary Figure 2. Effects of Rb overexpression on CDK4/6-dependent cell-cycle entry.

a Immunoblot showing endogenous and exogenous Rb expression in MCF-10A and RPE1 cells. **b**, **c** Representative density scatterplot showing Hoechst and EdU staining in MCF-10A (**b**) and RPE1 (**c**) cells without and with YFP-Rb expression (*n* = 2,000 cells/condition). **d** Percentage of each cell-cycle phase in MCF-10A and RPE1 cells without and with YFP-Rb expression. Data are shown as mean ± SD (*n* = 3 biological replicates). **e** Violin plots showing the distribution of YFP-Rb levels in MCF-10A and RPE1 cells 48 hr after DMSO treatment, contact inhibition, and mitogen removal (MCF-10A: control, *n* = 6,331 cells; CI, *n* = 8,638 cells; mitogen removal, *n* = 1,018 cells; RPE1: *n* = 2,500 cells/condition). **f** Average YFP-Rb levels in MCF-10A and RPE1 cells in the indicated conditions. Data are shown as mean ± SD

(*n* = 3 biological replicates). Asterisks indicate significant differences in the one-way ANOVA test (**$p \leq 0.001$; ***$p \leq 0.0001$). **g, h** Heatmap of single-cell traces for H2B-iRFP670, YFP-Rb, and NLS-mRuby3 levels in MCF-10A cells treated with DMSO (**g**) or palbociclib (1 μM) (**h**). Each row represents a single-cell trace over time according to the respective color map. **i** Heatmap of single-cell traces for CDK2 activity and YFP-Rb level in MCF-10A cells expressing a doxycycline-inducible cyclin E1 construct. Cells were treated with palbociclib (1 μM). **j** Average traces of CDK2 activity and YFP-Rb level in MCF-10A cells treated with palbociclib (1 μM) + DMSO or doxycycline (5 μM). Data are shown as mean ± 95% CI (DMSO: *n* = 291 cells; doxycycline: *n* = 369 cells).

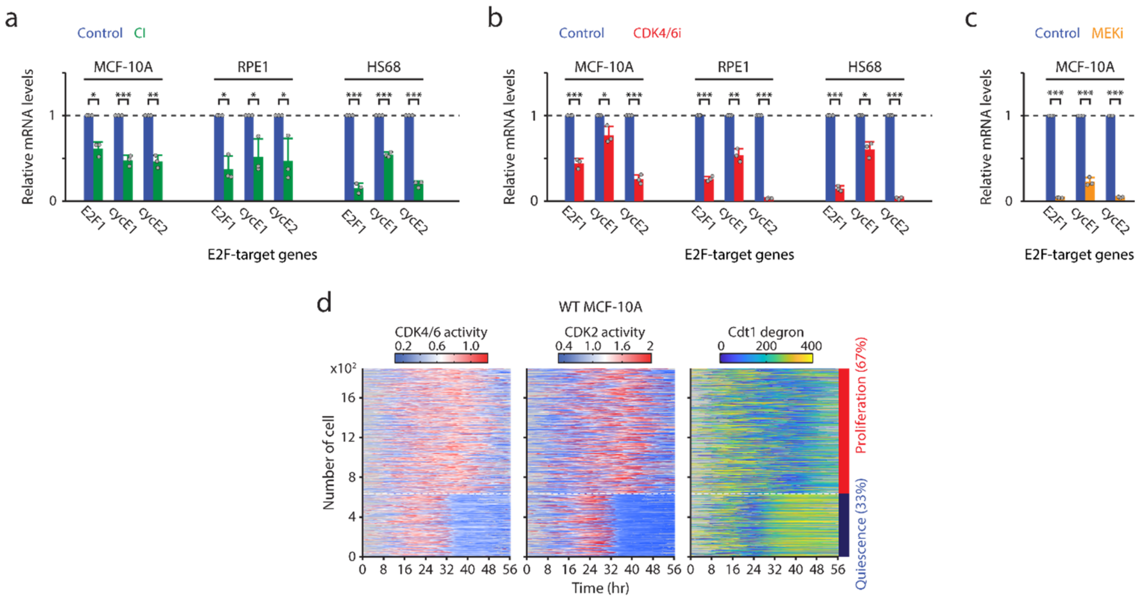

Supplementary Figure 3. Rb-protein reduction results in ineffective E2F activation.

a–c Relative mRNA levels of E2F-target genes in cells after contact inhibition (**a**) and treatment with palbociclib (1 μM) (**b**) or trametinib (10 nM) (**c**) for 48 hr. Data are shown as mean ± SD (n = 3 biological replicates). Asterisks indicate significant differences in the two-tailed unpaired t-test (*$p \leq 0.05$; **$p \leq 0.001$; ***$p \leq 0.0001$). **d** Heatmap of single-cell traces for CDK4/6 and CDK2 activities and Cdt1 degron level in wild-type MCF-10A cells. The percentages on the right of the panels indicate the proportion of proliferating and quiescent cells.

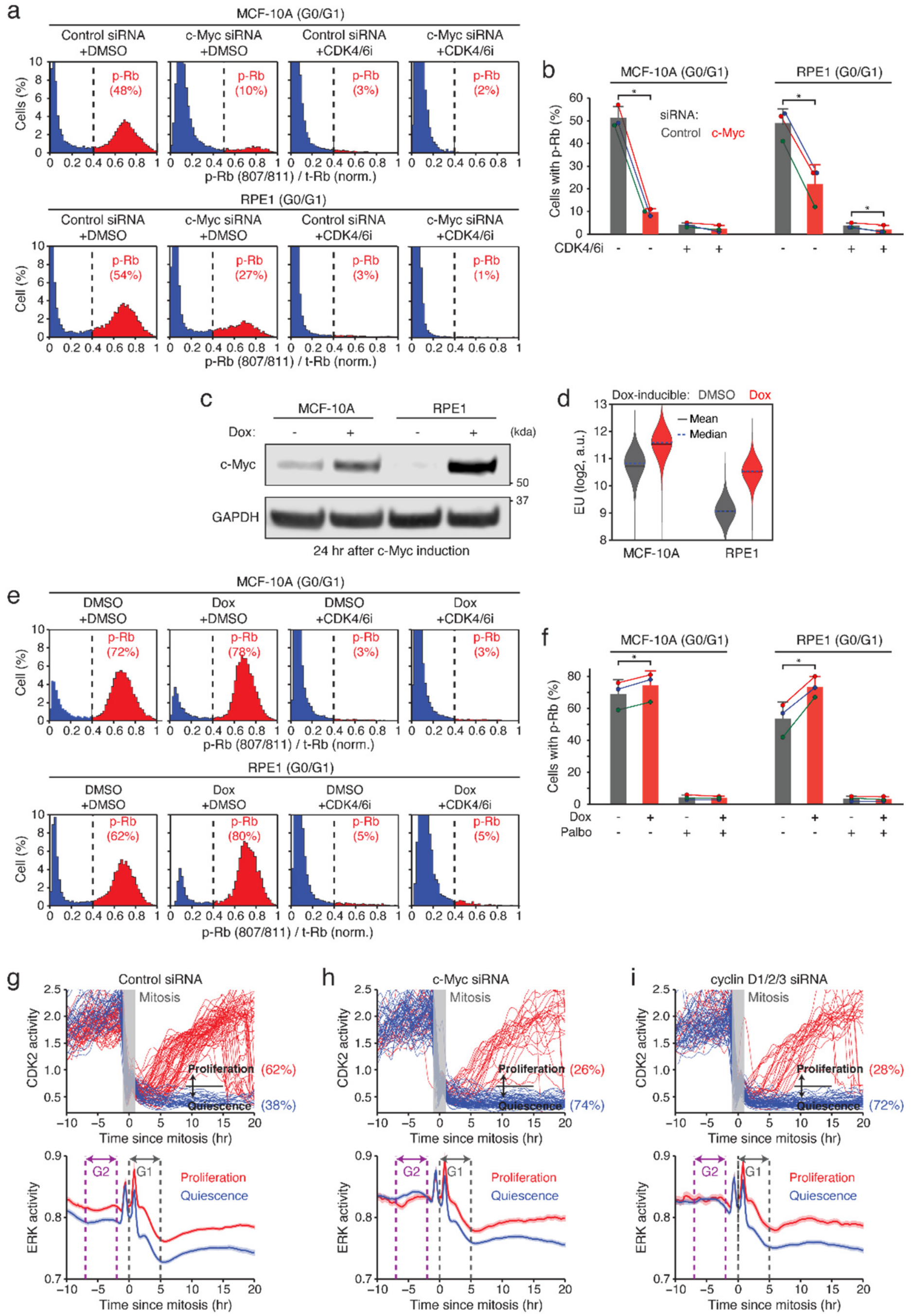

Supplementary Figure 4. c-Myc amplifies transcriptional activity and induces CDK4/6 activation, promoting cell proliferation.

a Representative histogram showing p-Rb (S807/811) normalized by t-Rb in G0/G1-phase MCF-10A and RPE1 cells. p-Rb negative and positive cells were displayed in blue and red, respectively. Following control or c-Myc siRNA knockdown for 48 hr, cells were treated with EdU (10 μM) + DMSO or palbociclib (1 μM) for 15 min prior to fixation (MCF-10A: control

siRNA + DMSO, *n* = 15,948 cells; c-Myc siRNA + DMSO, *n* = 9,298 cells; control siRNA + palbociclib, *n* = 16,381 cells; c-Myc siRNA + palbociclib, *n* = 4,465 cells; RPE1: control siRNA + DMSO, *n* = 11,959 cells; c-Myc siRNA + DMSO, *n* = 9,526 cells; control siRNA + palbociclib, *n* = 13,251 cells; c-Myc siRNA + palbociclib, *n* = 12,267 cells). **b** Percentage of p-Rb positive MCF-10A and RPE1 cells in G0/G1 phase 48 hr after control or c-Myc siRNA knockdown. Colored lines indicate paired-experimental set. Data are shown as mean ± SD (*n* = 3 biological replicates). Asterisks indicate significant differences in the two-tailed paired *t*-test (*$p \leq 0.05$). **c** Immunoblot showing c-Myc and GAPDH expression in MCF-10A and RPE1 cells expressing a doxycycline-inducible c-Myc construct after treatment with DMSO or doxycycline (5 μM) for 24 hr. **d** Violin plots showing distribution of EU level for MCF-10A and RPE1 cells after treatment with DMSO or doxycycline (5 μM) for 24 hr (*n* = 1,500 cells/condition). **e** Representative histogram showing p-Rb (S807/811) normalized by t-Rb in G0/G1-phase MCF-10A and RPE1 cells expressing a doxycycline-inducible c-Myc construct. Following DMSO or doxycycline (5 μM) treatment for 24 hr, cells were treated with EdU (10 μM) + DMSO or palbociclib (1 μM) for 15 min prior to fixation (MCF-10A: DMSO + DMSO, *n* = 17,409 cells; doxycycline + DMSO, *n* = 17,171 cells; DMSO + palbociclib, *n* = 16,968 cells; doxycycline + palbociclib, *n* = 17,110 cells; RPE1: DMSO + DMSO, *n* = 10,327 cells; doxycycline + DMSO, *n* = 5,697 cells; DMSO + palbociclib, *n* = 8,496 cells; doxycycline + palbociclib, *n* = 3,146 cells). **f** Percentage of p-Rb positive MCF-10A and RPE1 cells in G0/G1 phase. Cells were treated with DMSO or doxycycline (5 μM) for 24 hr. Colored lines indicate paired-experimental set. Data are shown as mean ± SD (*n* = 3 biological replicates). Asterisks indicate significant differences in the two-tailed paired *t*-test (*$p \leq 0.05$). **g–i** Single-cell traces of CDK2 activity aligned by mitosis in MCF-10A cells. Based on CDK2 activity (threshold = 0.7, black line) between 9 and 15 hr after mitosis, daughter cells were classified into proliferation or quiescence. Timelapse imaging was performed 6 hr after transfection with control (**g**), c-Myc (**h**), or cyclin D1/2/3 (**i**) siRNA. Average ERK activity traces aligned by mitosis and classified based on CDK2 activity. Data are shown as mean ± 95% CI (control siRNA: proliferation, *n* = 2,668 cells; quiescence, *n* = 1,629 cells; c-Myc siRNA: proliferation, *n* = 779 cells; quiescence, *n* = 2,187 cells; cyclin D1/2/3 siRNA: proliferation, *n* = 596 cells; quiescence, *n* = 1,563 cells).

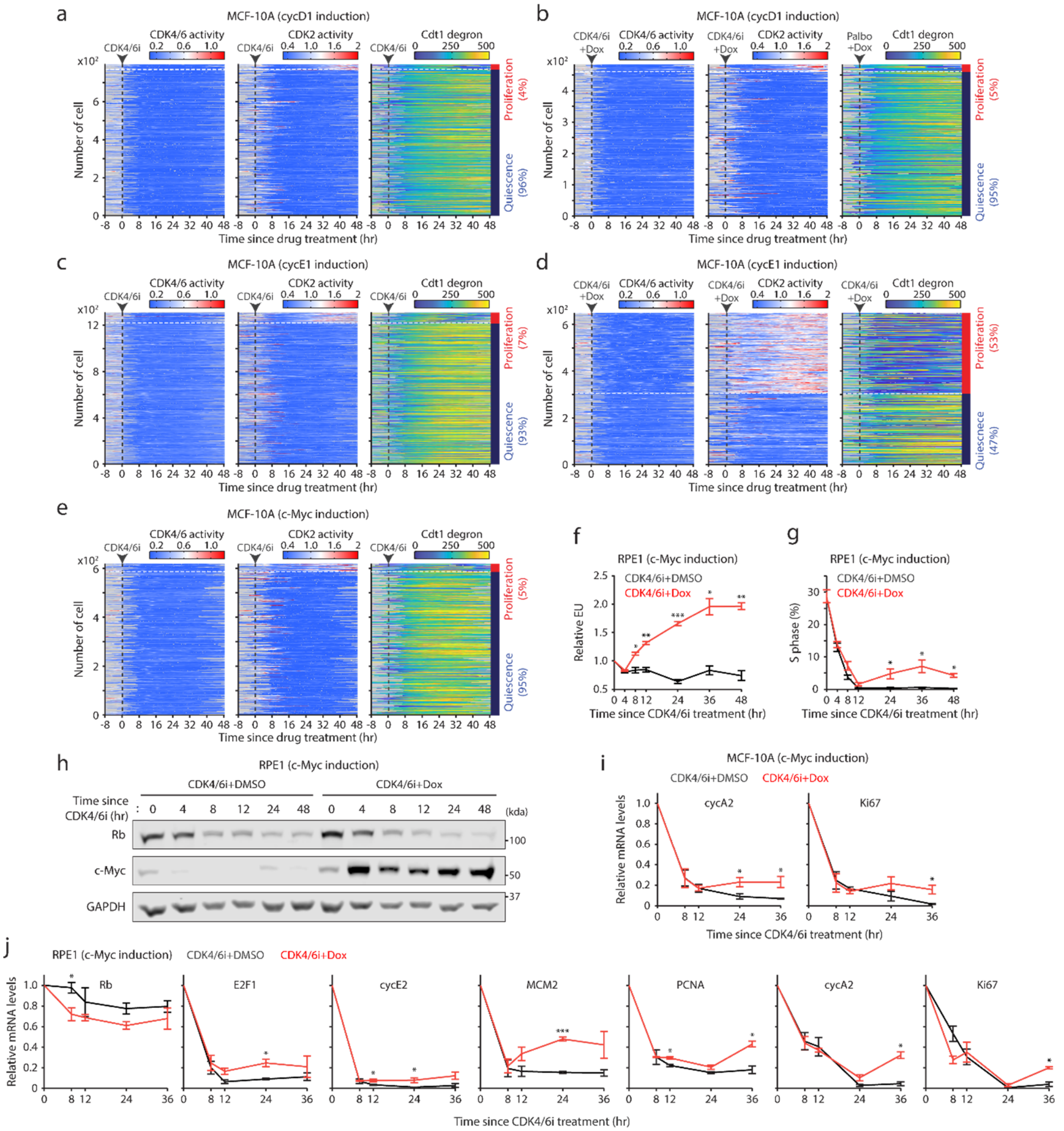

Supplementary Figure 5. c-Myc amplifies E2F transcriptional activity to induce cell proliferation without CDK4/6 activity.

a–**e** Heatmap of single-cell traces for CDK4/6 and CDK2 activities and Cdt1 degron level in tKO MCF-10A cells expressing a doxycycline-inducible cyclin D1 (**a**, **b**), cyclin E1 (**c**, **d**), or c-Myc (**e**) construct. Cells were treated with palbociclib (1 µM) alone (**a**, **c**, **e**) or palbociclib + doxycycline (5 µM) (**b**, **d**). **f**, Relative EU level in RPE1 cells. Cells were incubated with EU (1 mM) for 15 min prior to fixation. Data are shown as mean ± SEM (*n* = 3 biological replicates). **g** Percentage of S-phase cells in RPE1 cells. Cells were incubated with EdU (10 µM) for 15 min prior to fixation. Data are shown as mean ± SEM (*n* = 3 biological replicates). **h**

Immunoblot showing Rb, c-Myc, and GAPDH expression in RPE1 cells. **i**, **j** Relative mRNA level changes in two E2F-target genes in MCF-10A (i) and RPE1 (j) cells expressing a doxycycline-inducible c-Myc construct after treatment with palbociclib (1 μM) + DMSO or doxycycline (5 μM) for the indicated time (0–36 hr). Data are shown as mean ± SEM ($n = 3$ biological replicates). Asterisks indicate significant differences in the two-tailed unpaired *t*-test (*$p \leq 0.05$).

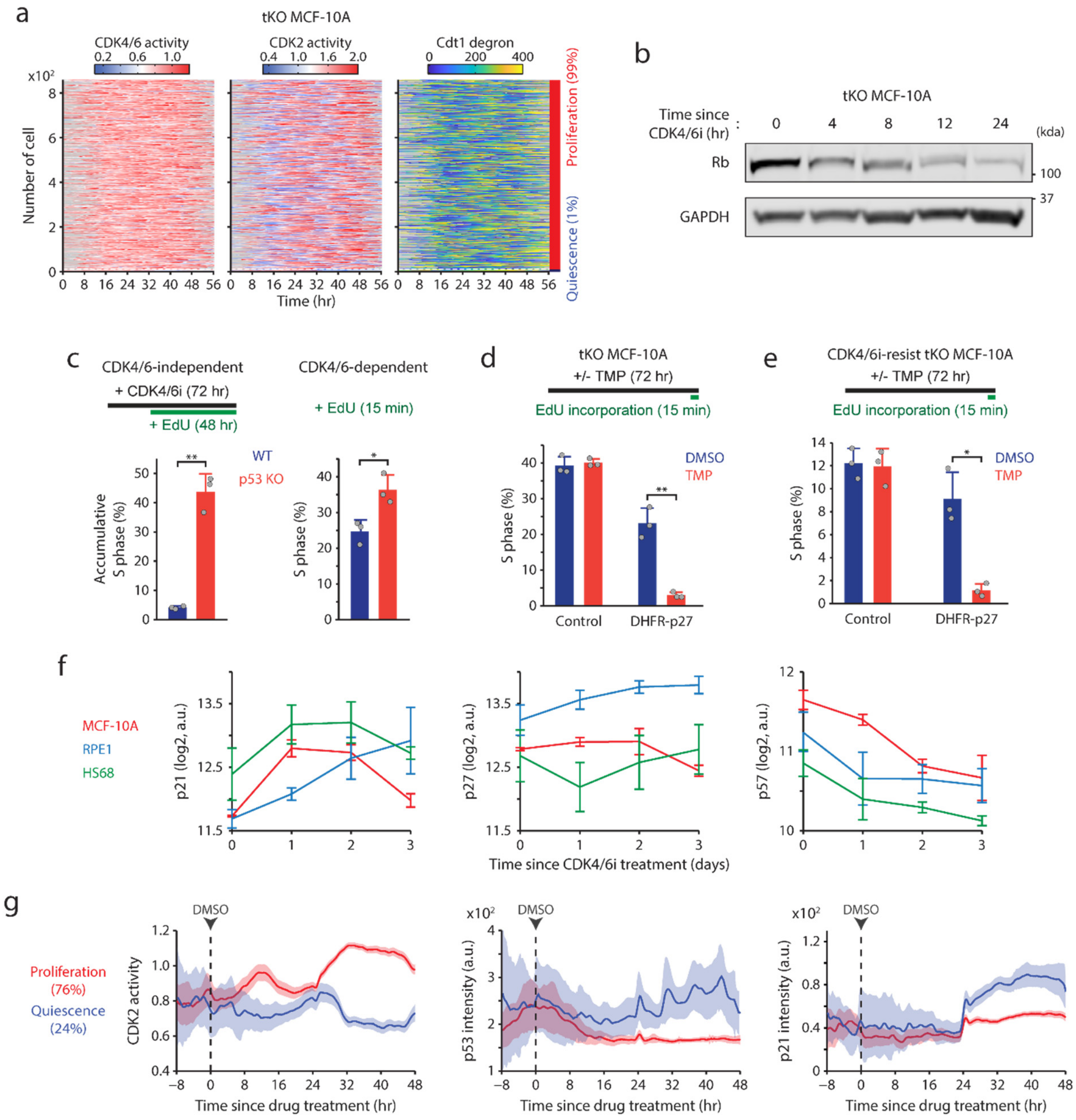

Supplementary Figure 6. Cip/Kip family proteins suppress both CDK4/6-dependent and -independent cell proliferation.

a Heatmap of single-cell traces for CDK4/6 and CDK2 activities and Cdt1 degron level in tKO MCF-10A cells. The percentages on the right of the panels indicate the proportion of proliferating and quiescent cells. **b** Immunoblot showing Rb and GAPDH expression in tKO MCF-10A cells after palbociclib (1 μM) treatment for the indicated time (0–24 hr). **c** Percentage of S-phase cells in wild-type and p53 knockout MCF-10A cells. 24 hr after palbociclib (1 μM) treatment, EdU (10 μM) was added for 48 hr to measure CDK4/6-independent cell proliferation. Cells were incubated with EdU (10 μM) for 15 min prior to

fixation to measure CDK4/6-dependent cell proliferation. Data are shown as mean ± SD (*n* = 3 biological replicates). Asterisks indicate significant differences in the two-tailed unpaired *t*-test (*$p \leq 0.05$; **$p \leq 0.001$). **d**, **e** Percentage of S-phase cells in wild-type (**d**) and palbociclib-resistant tKO (**e**) MCF-10A cells without and with expression of a DHFR-p27 construct. Cells were treated with either DMSO or TMP (50 μM) for 72 hr and incubated with EdU (10 μM) for 15 min prior to fixation. Data are shown as mean ± SD (*n* = 3 biological replicates). Asterisks indicate significant differences in the two-tailed unpaired *t*-test (*$p \leq 0.05$; **$p \leq 0.001$). **f** Expression changes in Cip/Kip levels in MCF-10A, RPE1, and HS68 cells treated with palbociclib (1 μM) for the indicated time (0–3 days). Data are shown as mean ± SEM (*n* = 3 biological replicates). **g** Average traces of CDK2 activity and p53 and p21 levels in MCF-7 cells treated with DMSO. Arrows and black dotted lines mark the drug-treatment time. Cells were classified based on CDK2 activity. Data are shown as mean ± 95% CI (proliferation: *n* = 1,392 cells; quiescence: *n* = 430 cells).

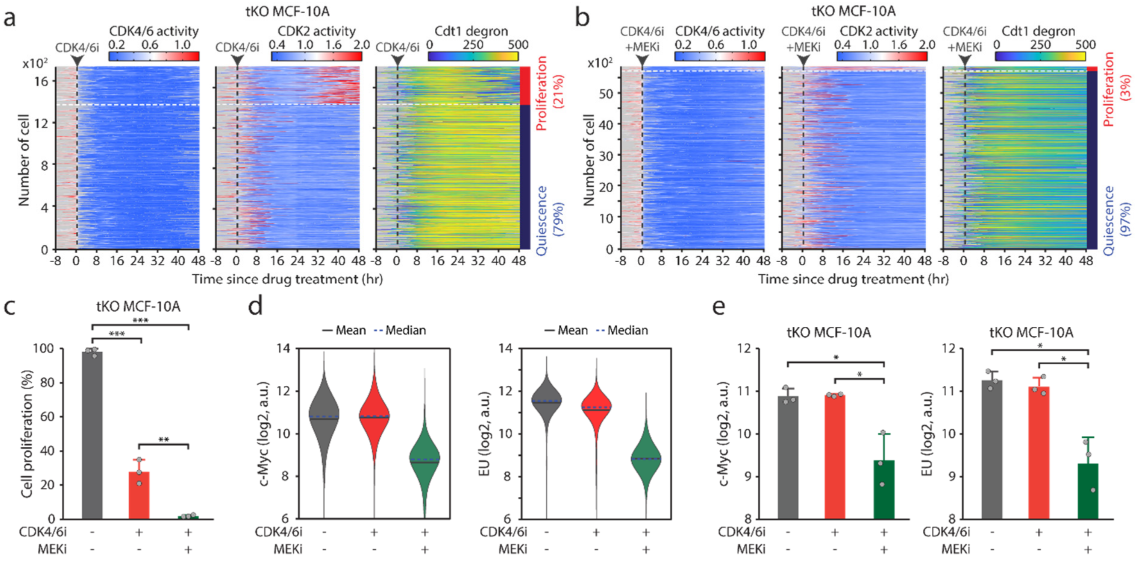

Supplementary Figure 7. Mitogenic signaling controls cell-cycle entry independent of CDK4/6 activity, preceding the involvement of Cip/Kip family proteins.

a, **b** Heatmap of single-cell traces for CDK4/6 and CDK2 activities and Cdt1 degron level in tKO MCF-10A cells. Cells were treated with palbociclib (1 µM) (**a**) or palbociclib + trametinib (100 nM) (**b**). **c** Percentage of proliferating tKO MCF-10A cells treated with DMSO, palbociclib (1 µM), or palbociclib + trametinib (100 nM). Data are shown as mean ± SD (*n* = 3 biological replicates). Asterisks indicate significant differences in the two-tailed unpaired *t*-test (***p* ≤ 0.001). **d** Violin plots showing distribution of c-Myc and EU levels in tKO MCF-10A cells treated with the indicated drug for 48 hr. Cells were incubated with EU (1 mM) for 30 min prior to fixation (*n* = 2,500 cells/condition). **e** Average c-Myc and EU levels in tKO MCF-10A cells treated with the indicated drug. Data are shown as mean ± SD (*n* = 3 biological replicates). Asterisks indicate significant differences in the two-tailed unpaired *t*-test (**p* ≤ 0.05).

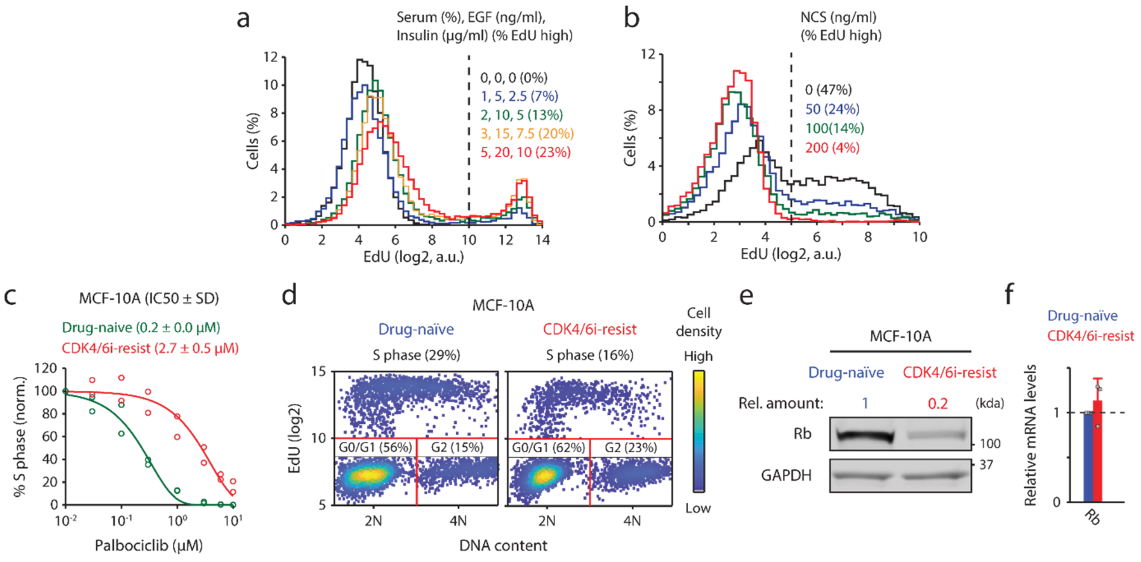

Supplementary Figure 8. Mitogenic and DNA damage signaling regulate CDK4/6-independent cell proliferation and validation of CDK4/6i-resistant MCF-10A cells.

a Representative histogram of EdU level in tKO MCF-10A cells showing classification of S-phase cells. 24 hr after treatment with palbociclib (1 µM), cells were incubated with EdU (10 µM), and different concentrations of mitogens as indicated for 48 hr prior to fixation (*n* > 11,000 cells/condition). **b** Representative histogram of EdU level in MCF-10A cells expressing a doxycycline-inducible c-Myc construct showing classification of S-phase cells. 24 hr after treatment with palbociclib (1 µM), cells were incubated with EdU (10 µM), doxycycline (5 µM), and various concentrations of NCS as indicated for 48 hr prior to fixation (*n* > 9,000 cells/condition). **c** Dose-response assay in drug-naive and palbociclib-resistant MCF-10A cells treated with different concentrations of palbociclib (0.01–10 µM) for 48 hr. IC50 values are shown as mean ± SD (*n* = 2 biological replicates). **d** Density scatterplot of Hoechst and EdU staining in drug-naive and palbociclib-resistant MCF-10A cells. Cells were incubated with EdU (10 µM) for 15 min prior to fixation (*n* = 5,000 cells/condition). **e** Immunoblot showing Rb and GAPDH expression in drug-naive and palbociclib-resistant MCF-10A cells. Relative amount indicates Rb-band intensities after normalizing with the loading control GAPDH. **f** Relative Rb mRNA levels. Data are shown as mean ± SD (*n* = 3 biological replicates).

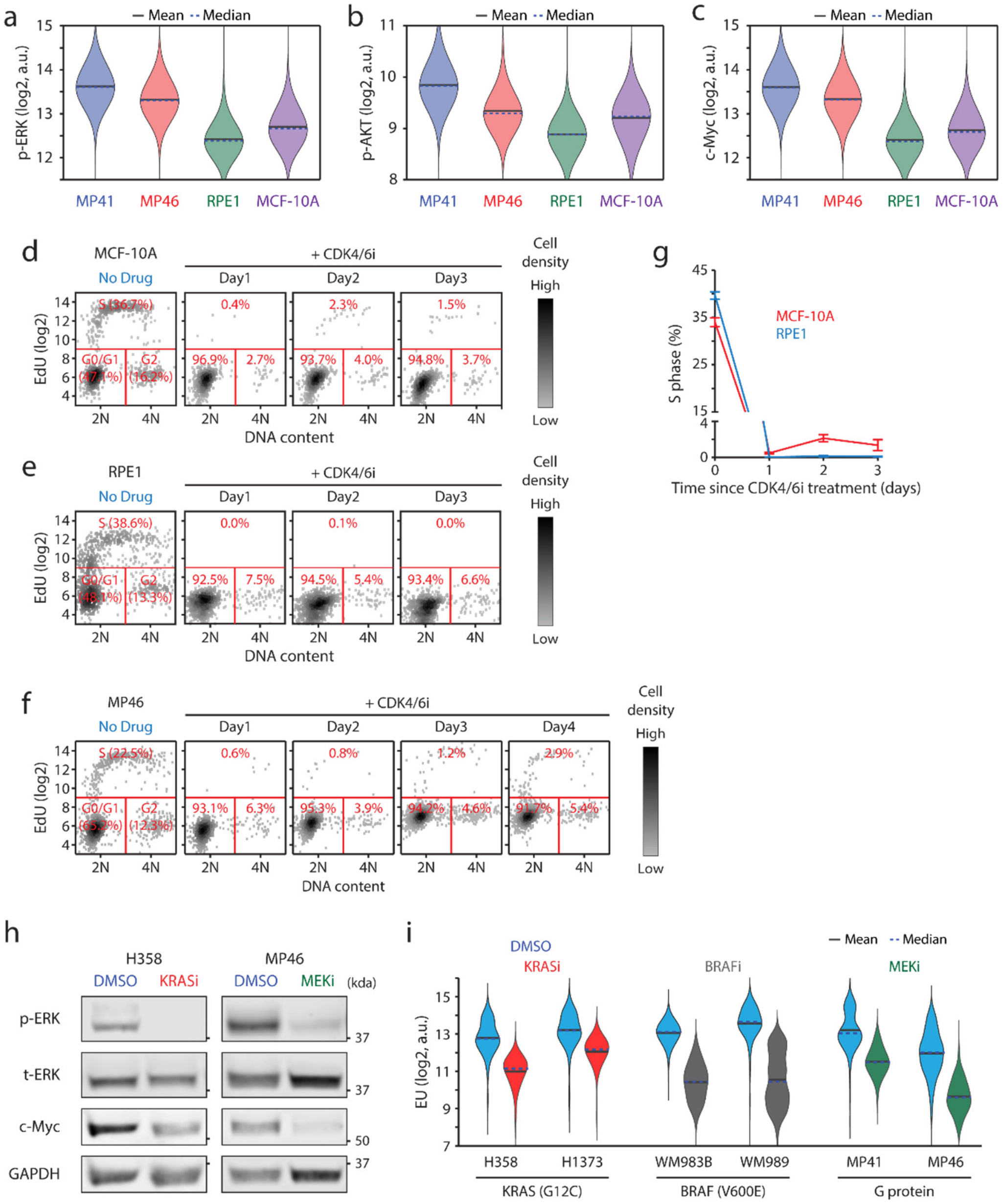

Supplementary Figure 9. **Activating mutations in mitogenic signaling in cancer facilitate adaptation to CDK4/6i.**

a–**c** Violin plots showing distribution of p-ERK (**a**), p-AKT (**b**), and c-Myc (**c**) levels in cancer and non-transformed cell lines ($n > 10{,}000$ cells/condition). **d**–**f** Density scatterplot of Hoechst and EdU staining in MCF-10A (**d**), RPE1 (**e**), and MP46 (**f**) cells treated with palbociclib (1 µM) for the indicated time. Cells were incubated with EdU (10 µM) for 15 min prior to fixation ($n = 1{,}000$ cells/condition). **g** Percentage of S-phase in MCF-10A and RPE1 cells treated with palbociclib (1 µM) for the indicated time. Data are shown as mean ± SEM (n = 3 biological

replicates). **h** Immunoblot showing p-ERK, t-ERK, c-Myc, and GAPDH expression in H358 and MP46 cells treated with the indicated drug for 24 hr (sotorasib, 1 μM; trametinib, 10 nM). **i** Violin plots showing the distribution of EU level in multiple cancer cell lines 24 hr after treatment with the indicated drug (sotorasib, 1 μM; vemurafenib, 1 μM; trametinib, 10 nM). Cells were incubated with EU (1 mM) for 30 min prior to fixation (*n* = 2,500 cells/condition).

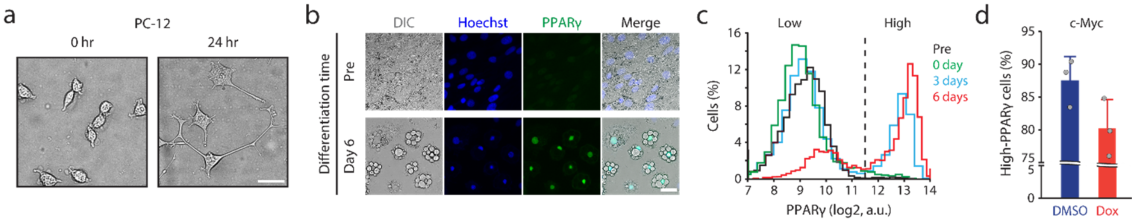

Supplementary Figure 10. **Validation of cell differentiation processes.**

a Representative differential interference contrast (DIC) images of undifferentiated and differentiated PC-12 cell appearance. The scale bar is 50 µm. **b** Representative DIC images, Hoechst, PPARγ, and merged images in OP-9 cells before and after 6-day differentiation. The scale bar is 50 µm. **c** Histogram of PPARγ level in OP-9 cells showing classification of high- and low-PPARγ expressing cells before and after differentiation for the indicated time. **d** Percentage of OP-9 cells with high-PPARγ. After 6 days of differentiation, cells expressing a doxycycline-inducible c-Myc construct were treated with palbociclib (1 µM), EdU (10 µM) + DMSO or doxycycline (5 µM) for 72 hr. Data are shown as mean ± SD (n = 3 biological replicates).

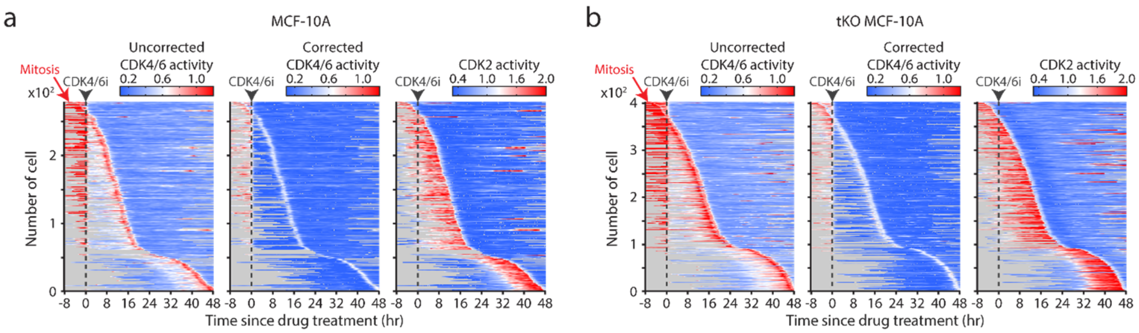

Supplementary Figure 11. **Calibration of non-specific signals in the CDK4/6 reporter during S/G2 phases based on the CDK2 reporter.**

a, **b** Heatmap of single-cell traces for uncorrected and corrected CDK4/6 activity and CDK2 activity sorted by the time of mitosis in wild-type (**a**) and tKO (**b**) MCF-10A cells. Cells were treated with palbociclib (1 μM).
