# Supplementary material for: Non-canonical pathway for Rb inactivation and external signaling coordinate cell-cycle entry without CDK4/6 activity
Source: Nat Commun. 2023 Nov 29;14:7847. doi: 10.1038/s41467-023-43716-y (PMC10687137; doi:10.1038/s41467-023-43716-y)
Supplement: Supplementary file 3 — Description of Additional Supplementary Files [file 41467_2023_43716_MOESM3_ESM.pdf]

File name: Supplementary Data 1

Description: Additional Statistical Information
