# Supplementary material for: Non-canonical pathway for Rb inactivation and external signaling coordinate cell-cycle entry without CDK4/6 activity
Source: Nat Commun. 2023 Nov 29;14:7847. doi: 10.1038/s41467-023-43716-y (PMC10687137; doi:10.1038/s41467-023-43716-y)
Supplement: Supplementary file 4 — Supplementary Data 1 [file 41467_2023_43716_MOESM4_ESM.docx]

| **Panel** | **Statistical test** | **P value** | **Test statistics** |
| --- | --- | --- | --- |
| Fig. 1g | One-way ANOVA with Tukey’s post hoc analysis | **MCF-10A** Rb:  0 day vs 1 day: P = 0.0012  0 day vs 2 day: P = 0.0016  1 day vs 2 day: P = 0.9780  E2F1:  0 day vs 1 day: P = 0.0026  0 day vs 2 day: P = 0.0017  1 day vs 2 day: P = 0.9470  cycE1:  0 day vs 1 day: P = 0.0395  0 day vs 2 day: P = 0.0189  1 day vs 2 day: P = 0.8846  cycE2:  0 day vs 1 day: P = 0.0063  0 day vs 2 day: P = 0.0578  1 day vs 2 day: P = 0.3561  **RPE1**  Rb:  0 day vs 1 day: P = 0.0464  0 day vs 2 day: P = 0.0752  1 day vs 2 day: P = 0.9486  E2F1:  0 day vs 1 day: P = 0.0008  0 day vs 2 day: P = 0.0084  1 day vs 2 day: P = 0.2438  cycE1:  0 day vs 1 day: P = 0.0006  0 day vs 2 day: P = 0.0033  1 day vs 2 day: P = 0.4274  cycE2:  0 day vs 1 day: P = 0.0002  0 day vs 2 day: P = 0.0040  1 day vs 2 day: P = 0.0986  **HS68**  Rb:  0 day vs 1 day: P = 0.1041  0 day vs 2 day: P = 0.1844  1 day vs 2 day: P = 0.9231  E2F1:  0 day vs 1 day: P = 0.0423  0 day vs 2 day: P = 0.2642  1 day vs 2 day: P = 0.4274  cycE1:  0 day vs 1 day: P = 0.0463  0 day vs 2 day: P = 0.2923  1 day vs 2 day: P = 0.4630  cycE2:  0 day vs 1 day: P = 0.0044  0 day vs 2 day: P = 0.0997  1 day vs 2 day: P = 0.1535 | **MCF-10A**  Rb:  0 day vs 1 day: 95% CI = [-1.313, -0.4118]  0 day vs 2 day: 95% CI = [-1.281, -0.3793]  1 day vs 2 day: 95% CI = [-0.4182, 0.4832]  E2F1:  0 day vs 1 day: 95% CI = [-6.908, -1.817]  0 day vs 2 day: 95% CI = [-7.196, -2.104]  1 day vs 2 day: 95% CI = [-2.833, 2.258]  cycE1:  0 day vs 1 day: 95% CI = [-4.950, -0.1298]  0 day vs 2 day: 95% CI = [-5.360, -0.5398]  1 day vs 2 day: 95% CI = [-2.820, 2.000]  cycE2:  0 day vs 1 day: 95% CI = [-11.88, -2.328]  0 day vs 2 day: 95% CI = [-9.394, 0.1593]  1 day vs 2 day: 95% CI = [-2.289, 7.264]  **RPE1**  Rb:  0 day vs 1 day: 95% CI = [-1.180, -0.01012]  0 day vs 2 day: 95% CI = [-1.115, 0.05488]  1 day vs 2 day: 95% CI = [-0.5199, 0.6499]  E2F1:  0 day vs 1 day: 95% CI = [-8.723, -2.982]  0 day vs 2 day: 95% CI = [-6.935, -1.195]  1 day vs 2 day: 95% CI = [-1.083, 4.658]  cycE1:  0 day vs 1 day: 95% CI = [-4.233, -1.517]  0 day vs 2 day: 95% CI = [-3.598, -0.8816]  1 day vs 2 day: 95% CI = [-0.7234, 1.993]  cycE2:  0 day vs 1 day: 95% CI = [-8.481, -3.559]  0 day vs 2 day: 95% CI = [-6.406, -1.484]  1 day vs 2 day: 95% CI = [-0.3863, 4.536]  **HS68**  Rb:  0 day vs 1 day: 95% CI = [-1.769, 0.1640]  0 day vs 2 day: 95% CI = [-1.637, 0.2965]  1 day vs 2 day: 95% CI = [-0.8340, 1.099]  E2F1:  0 day vs 1 day: 95% CI = [-34.19, -0.6398]  0 day vs 2 day: 95% CI = [-26.87, 6.678]  1 day vs 2 day: 95% CI = [-9.458, 24.09]  cycE1:  0 day vs 1 day: 95% CI = [-6.061, -0.05376]  0 day vs 2 day: 95% CI = [-4.731, 1.276]  1 day vs 2 day: 95% CI = [-1.674, 4.334]  cycE2:  0 day vs 1 day: 95% CI = [-26.42, -5.928]  0 day vs 2 day: 95% CI = [-18.86, 1.634]  1 day vs 2 day: 95% CI = [-2.684, 17.81] |
| Fig. 1i | Two-tailed unpaired Student’s *t*-test | **MCF-10A**  ACTB: P = 0.9170  Rb: P = 0.35  E2F1: P = 0.0002  CDC25A: P < 0.0001  **RPE1**  ACTB: P = 0.3760  Rb: P = 0.0912  E2F1: P = 0.015  CDC25A: P = 0.0138  **HS68**  ACTB: P = 0.6039  Rb: P = 0.0671  E2F1: P = 0.0135  CDC25A: P = 0.0059 | **MCF-10A**  ACTB: F = Infinity, P < 0.0001;  t = 0.1109, df = 4,  95% CI = [-0.2404, 0.2604]  Rb: F = Infinity, P < 0.0001;  t = 1.057, df = 4,  95% CI = [-0.1192, 0.2659]  E2F1: F = Infinity, P < 0.0001;  t = 13.29, df = 4,  95% CI = [1.527, 2.333]  CDC25A: F = Infinity, P < 0.0001;  t = 24.08, df = 4,  95% CI = [2.173, 2.740]  **RPE1**  ACTB: F = Infinity, P < 0.0001;  t = 0.9952, df = 4,  95% CI = [-0.1491, 0.3158]  Rb: F = Infinity, P < 0.0001;  t = 2.214, df = 4,  95% CI = [-0.02626, 0.2329]  E2F1: F = Infinity, P < 0.0001;  t = 8.121, df = 4,  95% CI = 0.7458, 1.521]  CDC25A: F = Infinity, P < 0.0001;  t = 4.195, df = 4  95% CI = [0.7214, 3.545]  **HS68**  ACTB: F = Infinity, P < 0.0001;  t = 0.5624, df = 4, 95% CI = -0.3806, 0.5739]  Rb: F = Infinity, P < 0.0001;  t = 2.495, df = 4, 95% CI = [-0.02517, 0.4718]  E2F1: F = Infinity, P < 0.0001;  t = 4.221, df = 4, 95% CI = 1.011, 4.896]  CDC25A: F = Infinity, P < 0.0001;  t = 12.91, df = 4, 95% CI = [3.888, 6.019] |
| Fig. 4c | Two-tailed unpaired Student’s *t*-test | MCF-10A: P < 0.0001  RPE1: P = 0.0003 | MCF-10A: F = Infinity, P < 0.0001;  t = 34.17, df = 4, 95% CI = [-0.9299, -0.7901]  RPE1: F = Infinity, P < 0.0001;  t = 12.25, df = 4, 95% CI = [-0.6093, -0.3841] |
| Fig. 4d | Two-tailed unpaired Student’s *t*-test | MCF-10A: P = 0.1283  RPE1: P = 0.0055 | MCF-10A: F = Infinity, P < 0.0001;  t = 1.913, df = 4, 95% CI = [-0.1369, 0.7436]  RPE1: F = Infinity, P < 0.0001;  t = 5.44, df = 4, 95% CI = [0.5435, 1.677] |
| Fig. 4f | One-way ANOVA with Tukey’s post hoc analysis | **Cyclin D1 induction** Palbo vs Palbo + Dox: P = 0.9447 Palbo vs Palbo + Tram: P = 0.2416 Palbo vs Palbo + Tram + Dox: P = 0.2096 Palbo + Dox vs Palbo + Tram: P = 0.1144 Palbo + Dox vs Palbo + Tram + Dox: P = 0.0984  Palbo + Tram vs Palbo + Tram + Dox: P = 0.9995  **Cyclin E1 induction**  Palbo vs Palbo + Dox: P = 0.0017  Palbo vs Palbo + Tram: P = 0.5254  Palbo vs Palbo + Tram + Dox: P = 0.8916  Palbo + Dox vs Palbo + Tram: P = 0.0004  Palbo + Dox vs Palbo + Tram + Dox: P = 0.0008  Palbo + Tram vs Palbo + Tram + Dox: P = 0.8916  **c-Myc induction**  Palbo vs Palbo + Dox: P = 0.0144  Palbo vs Palbo + Tram: P = 0.8292  Palbo vs Palbo + Tram + Dox: P = 0.9874  Palbo + Dox vs Palbo + Tram: P = 0.0049  Palbo + Dox vs Palbo + Tram + Dox: P = 0.0095  Palbo + Tram vs Palbo + Tram + Dox: P = 0.9500 | **Cyclin D1 induction** Palbo vs Palbo + Dox: CI = [-3.817, 2.703] Palbo vs Palbo + Tram: CI = [-1.155, 5.366] Palbo vs Palbo + Tram + Dox: CI = [-1.045, 5.475] Palbo + Dox vs Palbo + Tram: CI = [-0.5976, 5.923]  Palbo + Dox vs Palbo + Tram + Dox: CI = [-0.4883, 6.032]  Palbo + Tram vs Palbo + Tram + Dox: CI = [-3.151, 3.370]  **Cyclin E1 induction**  Palbo vs Palbo + Dox: CI = [-52.99, -15.43]  Palbo vs Palbo + Tram: CI = [-10.49, 27.07]  Palbo vs Palbo + Tram + Dox: CI = [-14.63, 22.92]  Palbo + Dox vs Palbo + Tram: CI = [23.72, 61.28]  Palbo + Dox vs Palbo + Tram + Dox: CI = [19.57, 57.13]  Palbo + Tram vs Palbo + Tram + Dox: CI = [-22.93, 14.63]  **c-Myc induction**  Palbo vs Palbo + Dox: CI = [-43.09, -5.330]  Palbo vs Palbo + Tram: CI = [-13.86, 23.89]  Palbo vs Palbo + Tram + Dox: CI = [-16.97, 20.78]  Palbo + Dox vs Palbo + Tram: CI = [10.35, 48.10]  Palbo + Dox vs Palbo + Tram + Dox: CI = [7.237, 44.99]  Palbo + Tram vs Palbo + Tram + Dox: CI = [-21.99, 15.77] |
| Fig. 4g | Two-tailed unpaired Student’s *t*-test | 4hr: P = 0.5375  8hr: P = 0.0008  12hr: P = 0.0029  24hr: P = 0.0009  36hr: P = 0.016  48hr: P = 0.0051 | 4hr: F = 3.0, P = 0.5;  t = 0.6736, df = 4, 95% CI = [-0.2185, 0.3585]  8hr: F = 2.036, P = 0.6588;  t = 9.22, df = 4, 95% CI = [0.1980, 0.3687]  12hr: F = 3.066, P = 0.4919;  t = 6.498, df = 4, 95% CI = [0.2386, 0.5947]  24hr: F = 10.33, P = 0.1765;  t = 8.752, df = 4, 95% CI = [0.4188, 0.8079]  36hr: F = 6.358, P = 0.2718;  t = 4.02, df = 4, 95% CI = [0.1701 to 0.9299]  48hr: F = 0.84, P = 0.91;  t = 5.569, df = 4, 95% CI = [0.2123 to 0.6344] |
| Fig. 4h | Two-tailed unpaired Student’s *t*-test | 4hr: P = 0.0232  8hr: P = 0.76  12hr: P = 0.0319  24hr: P = 0.0012  36hr: P = 0.0003  48hr: P = 0.0186 | 4hr: F = 2.896, P = 0.5133;  t = 3.579, df = 4, 95% CI = [-2.107, -0.2662]  8hr: F = 1.779, P = 0.7196;  t = 0.3220, df = 4, 95% CI = [-2.414, 3.047]  12hr: F = 6.899, P = 0.2532  t = 3.231, df = 4, 95% CI = [0.2420, 3.198]  24hr: F = 6629, P = 0.0003  t = 8.258, df = 4, 95% CI = [9.050, 18.22]  36hr: F = 2.084, P = 0.6486;  t = 11.81, df = 4, 95% CI = [4.074, 6.579]  48hr: F = 9.744, P = 0.1862;  t = 3.833, df = 4, 95% CI = [1.773, 11.09] |
| Fig. 4j | Two-tailed unpaired Student’s *t*-test | **Rb**  8hr: P = 0.0978  12hr: P = 0.0522  24hr: P = 0.2592  36hr: P = 0.3126  **E2F1**  8hr: P = 0.0775  12hr: P = 0.027  24hr: P = 0.1337  36hr: P = 0.0304  **cycE2**  8hr: P = 0.0232  12hr: P = 0.0093  24hr: P = 0.1843  36hr: P = 0.0269  **MCM2**  8hr: P = 0.0342  12hr: P = 0.0065  24hr: P = 0.1368  36hr: P = 0.0338  **PCNA**  8hr: P = 0.6896  12hr: P = 0.3447  24hr: P = 0.1117  36hr: P = 0.0054 | **Rb**  8hr: F = 1.98, P = 0.6712;  t = 2.152, df = 4, 95% CI = [-0.1985, 0.02516]  12hr: F = 5.286, P = 0.3182;  t = 2.735, df = 4, 95% CI = [-0.3224, 0.002421]  24hr: F = 2.147, P = 0.6356;  t = 1.314, df = 4, 95% CI = [-0.3840, 0.1373]  36hr: F = 13.67, P = 0.1363;  t = 1.154, df = 4, 95% CI = [-0.3291, 0.1358]  **E2F1**  8hr: F = 1.333, P = 0.8571;  t = 2.362, df = 4, 95% CI = [-0.01461, 0.1813]  12hr: F = 8.048, P = 0.2211;  t = 3.41, df = 4, 95% CI = [0.02910, 0.2842]  24hr: F = 5.694, P = 0.2988;  t = 1.877, df = 4, 95% CI = [-0.05428, 0.2809]  36hr: F = 2.053, P = 0.6552;  t = 3.283, df = 4, 95% CI = [0.01285, 0.1538]  **cycE2**  8hr: F = 4, P = 0.4;  t = 3.578, df = 4, 95% CI = [0.005972, 0.04736]  12hr: F = 1.564, P = 0.78;  t = 4.7, df = 4, 95% CI = [0.06412, 0.2492]  24hr: F = 57, P = 0.0345;  t = 1.602, df = 4, 95% CI = [-0.1075, 0.4008]  36hr: F = 28.43, P = 0.068;  t = 3.414, df = 4, 95% CI = [0.03050, 0.2962]  **MCM2**  8hr: F = 1.12, P = 0.9434;  t = 3.159, df = 4, 95% CI = [0.009291, 0.1440]  12hr: F = 11.38, P = 0.1615;  t = 5.202, df = 4, 95% CI = [0.1026, 0.3374]  24hr: F = 1.373, P = 0.8428;  t = 1.858, df = 4, 95% CI = [-0.08738, 0.4407]  36hr: F = 6.687, P = 0.2602;  t = 3.173, df = 4, 95% CI = [0.02997, 0.4500]  **PCNA**  8hr: F = 4, P = 0.4;  t = 0.4297, df = 4, 95% CI = [-0.1092, 0.1492]  12hr: F = 2.598, P = 0.5559;  t = 1.071, df = 4, 95% CI = [-0.1062, 0.2396]  24hr: F = 5.123, P = 0.3266;  t = 2.034, df = 4, 95% CI = [-0.04623, 0.2996]  36hr: F = 3.571, P = 0.4375;  t = 5.48, df = 4, 95% CI = [0.05098, 0.1557] |
| Fig. 5b | Two-tailed unpaired Student’s *t*-test | DMSO: P = 0.0003  CDK4/6i: P = 0.046 | DMSO: F = 3.044, P = 0.4945;  t = 11.36, df = 4, 95% CI = [20.26, 33.37]  CDK4/6i: F = 67.37, P = 0.0293;  t = 4.356, df = 4, 95% CI = [6.829, 30.83] |
| Fig. 5d | Two-tailed unpaired Student’s *t*-test | Palbo + DMSO: P = 0.580  Palbo + TMP: P = 0.0066 | Palbo + DMSO: F = 3.916, P = 0.2917;  t = 0.5848, df = 6, 95%, 95% CI = [-22.84, 14.03]  Palbo + TMP: F = 266.5, P = 0.0008;  t = 4.062, df = 6, 95% CI = [-61.11, -15.16] |
| Fig. 5f | One-way ANOVA with Tukey’s post hoc analysis | **Condition#**  1 vs 2: P = 0.5393  1 vs 3: P = 0.0036  1 vs 4: P = 0.0004  1 vs 5: P < 0.0001 2 vs 3: P = 0.0391  2 vs 4: P = 0.0036  2 vs 5: P = 0.0002  3 vs 4: P = 0.5393  3 vs 5: P = 0.0250  4 vs 5: P = 0.2701 | 1 vs 2: 95% CI = [-11.30, 3.965]  1 vs 3: 95% CI = [-19.30, -4.035]  1 vs 4: 95% CI = [-22.97, -7.701]  1 vs 5: 95% CI = [-27.97, -12.70] 2 vs 3: 95% CI = [-15.63, -0.3680]  2 vs 4: 95% CI = [-19.30, -4.035]  2 vs 5: 95% CI = [-24.30, -9.035]  3 vs 4: 95% CI = [-11.30, 3.965]  3 vs 5: 95% CI = [-16.30, -1.035]  4 vs 5: 95% CI = [-12.63, 2.632] |
| Fig. 5g | One-way ANOVA with Tukey’s post hoc analysis | **NCS (ng/ml)**  0 vs 50: P = 0.0716  0 vs 100: P = 0.0066  0 vs 200: P = 0.0014  50 vs 100: P = 0.3528  50 vs 200: P = 0.0595  100 vs 200: P = 0.5820 | 0 vs 50: 95% CI = [-1.294, 31.29]  0 vs 100: 95% CI = [7.706, 40.29]  0 vs 200: 95% CI = [14.37, 46.96]  50 vs 100: 95% CI = [-7.294, 25.29] 50 vs 200: 95% CI = [-0.6273, 31.96]  100 vs 200: 95% CI = [-9.627, 22.96] |
| Fig. 5h | Two-tailed unpaired Student’s *t*-test | 0 ng/ml NCS: P = 0.0418  50 ng/ml NCS: P = 0.0530  100 ng/ml NCS: P = 0.0183  2000 ng/ml NCS: P = 0.0077 | 0 ng/ml NCS: F = 1.781, P = 0.6470;  t = 2.579, df = 6, 95% CI = [0.2949, 11.25]  50 ng/ml NCS: F = 17.41, P = 0.0423;  t = 2.404, df = 6, 95% CI = [-0.03920, 4.409]  100 ng/ml NCS: F = 23.81, P = 0.0271;  t = 3.213, df = 6, 95% CI = [0.6519, 4.818]  200 ng/ml NCS: F = 1.807, P = 0.639;  t = 3.929, df = 6, 95% CI = [1.076, 4.629] |
| Fig. 6d | Two-tailed unpaired Student’s *t*-test | H358: P = 0.022  H1373: P = 0.0639  WM983B: P = 0.0024  WM989: P = 0.0005  MP41: P = 0.0124  MP46: P = 0.043 | H358: F = 1.456, P = 0.8142;  t = 3.637, df = 4,  95% CI = [-2.622, -0.3517]  H1373: F = 8.939, P = 0.2012;  t = 2.541, df = 4,  95% CI = [-1.556, 0.06893]  WM983B: F = 5.438, P = 0.3106;  t = 6.795, df = 4,  95% CI = [-4.240, -1.780]  WM989: F = 1.283, P = 0.8761;  t = 10.31, df = 4,  95% CI = [-4.709, -2.711]  MP41: F = 15.87, P = 0.1185  t = 4.33, df = 4,  95% CI = [-2.112, -0.4616]  MP46: F = 1.116, P = 0.9452  t = 2.915, df = 4  95% CI = [-2.812, -0.06838] |
| Fig. 6f | One-way ANOVA with Tukey’s post hoc analysis | **H1373**  DMSO vs Palbo: P <0.0001  DMSO vs mutation-specific drug: P = 0.0002  DMSO vs Palbo + drug: P <0.0001  Palbo + mutation-specific drug: P = 0.0101  Palbo vs Palbo + drug: P <0.0001  Mutation-specific drug vs Palbo + drug: P <0.0001  **WM989**  DMSO vs Palbo: P <0.0001  DMSO vs mutation-specific drug: P <0.0001  DMSO vs Palbo + drug: P <0.0001  Palbo + mutation-specific drug: P = 0.1121  Palbo vs Palbo + drug: P = 0.1160  Mutation-specific drug vs Palbo + drug: P = 0.0035  **MP41**  DMSO vs Palbo: P <0.0001  DMSO vs mutation-specific drug: P = 0.0096  DMSO vs Palbo + drug: P <0.0001  Palbo + mutation-specific drug: P = 0.0006  Palbo vs Palbo + drug: P = 0.5601  Mutation-specific drug vs Palbo + drug: P = 0.0002 | **H1373**  DMSO vs Palbo: 95% CI = [20.74, 35.58]  DMSO vs mutation-specific drug: 95% CI = [10.61, 25.44]  DMSO vs Palbo + drug: 95% CI = [44.84, 59.67]  Palbo + mutation-specific drug: 95% CI = [-17.55, -2.720]  Palbo vs Palbo + drug: 95% CI = [16.68, 31.51]  Mutation-specific drug vs Palbo + drug: 95% CI = [26.81, 41.65]  **WM989**  DMSO vs Palbo: 95% CI = [47.32, 70.12]  DMSO vs mutation-specific drug: 95% CI = [37.96, 60.76]  DMSO vs Palbo + drug: 95% CI = [56.60, 79.40]  Palbo + mutation-specific drug: 95% CI = [-20.76, 2.037]  Palbo vs Palbo + drug: 95% CI = [-2.124, 20.68]  Mutation-specific drug vs Palbo + drug: 95% CI = [7.239, 30.04]  **MP41**  DMSO vs Palbo: 95% CI = [27.44, 49.11]  DMSO vs mutation-specific drug: 95% CI = [4.101, 25.77]  DMSO vs Palbo + drug: 95% CI = [32.01, 53.67]  Palbo + mutation-specific drug: 95% CI = [-34.17, -12.51]  Palbo vs Palbo + drug: 95% CI = [-6.265, 15.40]  Mutation-specific drug vs Palbo + drug: 95% CI = [17.07, 38.74] |
| Fig. 7a | Two-tailed unpaired Student’s *t*-test and One-way ANOVA with Tukey’s post hoc analysis | **PLB-985**  0day vs 3day: P = 0.0004  0day vs 6day: P < 0.0001  3day vs 6day: P = 0.0291  **PC-12**  0day vs 1day: P = 0.0004  **OP-9**  Pre vs 0day: P < 0.0001  Pre vs 3day: P < 0.0001  Pre vs 6day: P < 0.0001  0day vs 3day: P = 0.9147  0day vs 6day: P = 0.9228  3day vs 6day: P >0.9999 | **PLB-985**  0day vs 3day: 95% CI = [21.83, 48.05]  0day vs 6day: 95% CI = [36.86, 63.08]  3day vs 6day: 95% CI = [1.922, 28.14]  **PC-12**  0day vs 1day: F = 1.3, P = 0.8694  t = 10.70, df = 4,  95% CI = [-25.06 to -14.74]  **OP-9**  Pre vs 0day: 95% CI = [23.19, 38.13]  Pre vs 3day: 95% CI = [24.69, 39.64]  Pre vs 6day: 95% CI = [24.63, 39.58]  0day vs 3day: 95% CI = [-5.970, 8.976]  0day vs 6day: 95% CI = [-6.026, 8.920]  3day vs 6day: 95% CI = [-7.530, 7.416] |
| Fig. 7b | Two-tailed unpaired Student’s *t*-test | P = 0.0069 | F = 32.50, P = 0.0597;  t = 5.116, df = 4,  95% CI= [16.11, 54.33] |
| Fig. 7c | One-way ANOVA with Tukey’s post hoc analysis | Pre vs 0day: P = 0.9942  Pre vs 3day: P = 0.0244  Pre vs 6day: P = 0.0009  0day vs 3day: P = 0.0344  0day vs 6day: P = 0.0012  3day vs 6day: P = 0.0992 | Pre vs 0day: 95% CI = [-37.84, 32.39]  Pre vs 3day: 95% CI = [-75.89, -5.659]  Pre vs 6day: 95% CI = [-105.7, -35.45]  0day vs 3day: 95% CI = [-73.16, -2.933]  0day vs 6day: 95% CI = [-103.0, -32.72]  3day vs 6day: 95% CI = [-64.90, 5.324] |
| Fig. 7e | Two-tailed unpaired Student’s *t*-test | P = 0.0020 | F = 1.658, P = 0.7525;  t = 7.164, df = 4,  95% CI= [6.051, 13.71] |
| Fig. S1c | Two-tailed unpaired Student’s *t*-test | **MCF-10A**  Rb: P = 0.0165  **RPE1**  Rb: P = 0.0073  **HS68**  Rb: P = 0.0057 | **MCF-10A**  Rb: F = Infinity, P < 0.0001;  t = 3.970, df = 4, 95% CI = [-0.4283, -0.07576]  **RPE1**  Rb: F = Infinity, P < 0.0001;  t = 5.041, df = 4, 95% CI = [-0.5502, -0.1594]  **HS68**  Rb: F = Infinity, P < 0.0001;  t = 5.389, df = 4, 95% CI = [-0.5552, -0.1776] |
| Fig. S1d | Two-tailed unpaired Student’s *t*-test | **MCF-10A**  Rb: P = 0.6769  **RPE1**  Rb: P = 0.0164  **HS68**  Rb: P < 0.0001 | **MCF-10A**  Rb: F = Infinity, P < 0.0001;  t = 0.4487, df = 4, 95% CI = [-0.3191, 0.2303]  **RPE1**  Rb: F = Infinity, P < 0.0001;  t = 3.977, df = 4, 95% CI = [-0.2895, -0.05145]  **HS68**  Rb: F = Infinity, P < 0.0001;  t = 31.35, df = 4, 95% CI = [-0.4822, -0.4037] |
| Fig. S1e | Two-tailed unpaired Student’s *t*-test | P = 0.0017 | F = Infinity, P < 0.0001;  t = 7.538, df = 4, 95% CI = [-0.4907, -0.2265] |
| Fig. S1f | Two-tailed unpaired Student’s *t*-test | P = 0.0320 | F = Infinity, P < 0.0001;  t = 3.229, df = 2, 95% CI = [-0.3009, -0.02270] |
| Fig. S2f | One-way ANOVA with Tukey’s post hoc analysis | **MCF-10A**  Control vs CI: P = 0.0003  Control vs mitogen removal: P < 0.0001  CI vs mitogen removal: P = 0.0359  **RPE1**  Control vs CI: P = 0.0008  Control vs mitogen removal: P = 0.0005  CI vs mitogen removal: P = 0.7029 | **MCF-10A**  Control vs CI: 95% CI = [1.177, 2.463]  Control vs mitogen removal: 95% CI = [1.877, 3.163]  CI vs mitogen removal: 95% CI = [0.05688, 1.343]  **RPE1**  Control vs CI: 95% CI = [1.054, 2.593]  Control vs mitogen removal: 95% CI = [1.261, 2.799]  CI vs mitogen removal: 95% CI = [-0.5625, 0.9758] |
| Fig. S3a | Two-tailed unpaired Student’s *t*-test | **MCF-10A**  E2F1: P = 0.0011  cycE1: P = 0.0001  cycE2: P = 0.0002  **RPE1**  E2F1: P = 0.0019  cycE1: P = 0.0163  cycE2: P = 0.0244  **HS68**  E2F1: P < 0.0001  cycE1: P < 0.0001  cycE2: P < 0.0001 | **MCF-10A**  E2F1: F = Infinity, P < 0.0001;  t = 8.409, df = 4, 95% CI = [-0.5185, -0.2611]  cycE1: F = Infinity, P < 0.0001;  t = 14.84, df = 4, 95% CI = [-0.6250, -0.4279]  cycE2: F = Infinity, P < 0.0001;  t = 12.86, df = 4, 95% CI = [-0.6544, -0.4220]  **RPE1**  E2F1: F = Infinity, P < 0.0001;  t = 7.279, df = 4, 95% CI = [-0.8647, -0.3872]  cycE1: F = Infinity, P < 0.0001;  t = 3.987, df = 4, 95% CI = [-0.8271, -0.1480]  cycE2: F = Infinity, P < 0.0001;  t = 3.520, df = 4, 95% CI = [-0.9466, -0.1118]  **HS68**  E2F1: F = Infinity, P < 0.0001;  t = 25.82, df = 4, 95% CI = [-0.9404, -0.7577]  cycE1: F = Infinity, P < 0.0001;  t = 27.73, df = 4, 95% CI = [-0.5051, -0.4132]  cycE2: F = Infinity, P < 0.0001;  t = 39.23, df = 4, 95% CI = [-0.8607, -0.7470] |
| Fig. S3b | Two-tailed unpaired Student’s *t*-test | **MCF-10A**  E2F1: P < 0.0001  cycE1: P = 0.0167  cycE2: P < 0.0001  **RPE1**  E2F1: P < 0.0001  cycE1: P = 0.0004  cycE2: P < 0.0001  **HS68**  E2F1: P < 0.0001  cycE1: P = 0.0015  cycE2: P < 0.0001 | **MCF-10A**  E2F1: F = Infinity, P < 0.0001;  t = 16.97, df = 4, 95% CI = [-0.6513, -0.4681]  cycE1: F = Infinity, P < 0.0001;  t = 3.959, df = 4, 95% CI = [-0.3926, -0.06892]  cycE2: F = Infinity, P < 0.0001;  t = 24.43, df = 4, 95% CI = [-0.8312, -0.6616]  **RPE1**  E2F1: F = Infinity, P < 0.0001;  t = 44.95, df = 4, 95% CI = [-0.7857, -0.6943]  cycE1: F = Infinity, P < 0.0001;  t = 10.65, df = 4, 95% CI = [-0.5872, -0.3444]  cycE2: F = Infinity, P < 0.0001;  t = 128, df = 4, 95% CI = [-0.9947, -0.9526]  **HS68**  E2F1: F = Infinity, P < 0.0001;  t = 47.83, df = 4, 95% CI = [-0.9047, -0.8054]  cycE1: F = Infinity, P < 0.0001;  t = 7.720, df = 4, 95% CI = [-0.5389, -0.2538]  cycE2: F = Infinity, P < 0.0001;  t = 119.7, df = 4, 95% CI = [-0.9905, -0.9456] |
| Fig. S3c | Two-tailed unpaired Student’s *t*-test | **MCF-10A**  E2F1: P < 0.0001  cycE1: P < 0.0001  cycE2: P < 0.0001 | **MCF-10A**  E2F1: F = Infinity, P < 0.0001;  t = 562.6, df = 4, 95% CI = [-0.9624, -0.9530]  cycE1: F = Infinity, P < 0.0001;  t = 27.55, df = 4, 95% CI = [-0.8504, -0.6947]  cycE2: F = Infinity, P < 0.0001;  t = 257.6, df = 4, 95% CI = [-0.9625, -0.9420] |
| Fig. S4b | Two-tailed paired Student’s *t*-test | **MCF-10A**  CDK4/6i-: P = 0.0031  CDK4/6i+: P = 0.1296  **RPE1**  CDK4/6i-: P = 0.0018  CDK4/6i+: P = 0.0377 | **MCF-10A**  CDK4/6i-: t = 17.86, df = 2, 95% CI = [-51.71, -31.63]  CDK4/6i+: t = 2.5, df = 2, 95% CI = [-4.535, 1.202]  **RPE1**  CDK4/6i-: t = 23.38, df = 2, 95% CI = [-31.97, -22.03]  CDK4/6i+: t = 5.0, df = 2, 95% CI = [-3.101, -0.2324] |
| Fig. S4f | Two-tailed paired Student’s *t*-test | **MCF-10A**  CDK4/6i-: P = 0.0039  CDK4/6i+: P = 0.4226  **RPE1**  CDK4/6i-: P = 0.0187  CDK4/6i+: P = 0.4226 | **MCF-10A**  CDK4/6i-: t = 16.0, df = 2, 95% CI = [3.899, 6.768]  CDK4/6i+: t = 1.0, df = 2, 95% CI = [-1.768, 1.101]  **RPE1**  CDK4/6i-: t = 7.208, df = 2, 95% CI = [7.927, 31.41] CDK4/6i+: t = 1.0, df = 2, 95% CI = [-1.768, 1.101] |
| Fig. S5f | Two-tailed unpaired Student’s *t*-test | 4hr: P = 0.3739  8hr: P = 0.0059  12hr: P = 0.0006  24hr: P < 0.0001  36hr: P = 0.0023  48hr: P = 0.0003 | 4hr: F = 1.333, P = 0.8571;  t = 1.0, df = 4, 95% CI = [-0.04145, 0.08812]  8hr: F = 5.186, P = 0.3233;  t = 5.334, df = 4, 95% CI = [0.1391, 0.4409]  12hr: F = 2.279, P = 0.61;  t = 9.97, df = 4, 95% CI = [0.3391, 0.6009]  24hr: F = 1.099, P = 0.9528;  t = 19.20, df = 4, 95% CI = [0.8725, 1.167]  36hr: F = 3.430, P = 0.4514;  t = 6.941, df = 4, 95% CI = [0.6740, 1.573]  48hr: F = 2.223, P = 0.6206;  t = 11.78, df = 4, 95% CI = [0.9351, 1.512] |
| Fig. S5g | Two-tailed unpaired Student’s *t*-test | 4hr: P = 0.5982  8hr: P = 0.0723  12hr: P = 0.0944  24hr: P = 0.0437  36hr: P = 0.0274  48hr: P = 0.0016 | 4hr: F = 1.333, P = 0.6726;  t = 1.973, df = 4, 95% CI = [-3.485, 5.291]  8hr: F = 4.110, P = 0.3914;  t = 2.426, df = 4, 95% CI = [-0.5049, 7.505]  12hr: F = 16.76, P = 0.1126;  t = 2.183, df = 4, 95% CI = [-0.3277, 2.741]  24hr: F = 68.81, P = 0.0286;  t = 2.909, df = 4, 95% CI = [0.2024, 8.658]  36hr: F = 174.7, P = 0114;  t = 3.393, df = 4, 95% CI = [1.186, 11.85]  48hr: F = 231.2, P = 0.0086;  t = 7.637, df = 4, 95% CI = [2.578, 5.522] |
| Fig. S5i | Two-tailed unpaired Student’s *t*-test | **cycA2**  8hr: P = 0.9791  12hr: P > 0.9999  24hr: P = 0.0535  36hr: P = 0.0446  **Ki67**  8hr: P = 0.8577  12hr: P = 0.3837  24hr: P = 0.1939  36hr: P = 0.0305 | **cycA2**  8hr: F = 1.442, P = 0.8190;  t = 0.02793, df = 4, 95% CI = [-0.3347, 0.3280]  12hr: F = 2.333, P = 0.600;  t = 0.0, df = 4, 95% CI = [-0.1341, 0.1341]  24hr: F = 3.211, P = 0.4750;  t = 2.711, df = 4, 95% CI = [-0.003375, 0.2834]  36hr: F = 91.00, P = 0.0217;  t = 2.889, df = 4, 95% CI = [0.006247,0.3138]  **Ki67**  8hr: F = 1.165, P = 0.9239;  t = 0.1912, df = 4, 95% CI = [-0.3621, 0.3154]  12hr: F = 2.526, P = 0.5672;  t = 0.9774, df = 4, 95% CI = [-0.1024, 0.04909]  24hr: F = 1.829, P = 0.7069;  t = 1.559, df = 4, 95% CI = [-0.09626, 0.3429]  36hr: F = 163.0, P = 0.0122;  t = 3.280, df = 4, 95% CI = [0.02148, 0.2585] |
| Fig. S5j | Two-tailed unpaired Student’s *t*-test | **Rb**  8hr: P = 0.0317  12hr: P = 0.3598  24hr: P = 0.0614  36hr: P = 0.3760  **E2F1**  8hr: P = 0.9889  12hr: P = 0.0550  24hr: P = 0.0111  36hr: P = 0.4139  **cycE2**  8hr: P = 0.8933  12hr: P = 0.0437  24hr: P = 0.0282  36hr: P = 0.0585  **MCM2**  8hr: P = 0.8352  12hr: P = 0.1049  24hr: P = 0.0001  36hr: P = 0.1058  **PCNA**  8hr: P > 0.9999  12hr: P = 0.0125  24hr: P = 0.1012  36hr: P = 0.0058  **cycA2**  8hr: P = 0.9877  12hr: P = 0.6989  24hr: P = 0.0927  36hr: P = 0.0021  **Ki67**  8hr: P = 0.2880  12hr: P = 0.7422  24hr: P = 0.2099  36hr: P = 0.0026 | **Rb**  8hr: F = 1.467, P = 0.8106;  t = 3.239, df = 4, 95% CI = [-0.4767, -0.03668]  12hr: F = 20.58, P = 0.0927;  t = 1.033, df = 4, 95% CI = [-0.5407, 0.2474]  24hr: F = 2.085, P = 0.6482;  t = 2.579, df = 4, 95% CI = [-0.3392, 0.01251]  36hr: F = 3.280, P = 0.4673;  t = 0.9951, df = 4, 95% CI = [-0.4422, 0.2088]  **E2F1**  8hr: F = 1.626, P = 0.7615;  t = 0.01484, df = 4, 95% CI = [-0.6270, 0.6203]  12hr: F = 3.464, P = 0.4480;  t = 2.683, df = 4, 95% CI = [-0.003472, 0.2035]  24hr: F = 34.33, P = 0.0566;  t = 4.468, df = 4, 95% CI = [0.05805, 0.2486]  36hr: F = 7.750, P = 0.2286;  t = 0.9108, df = 4, 95% CI = [-0.2048, 0.4048]  **cycE2**  8hr: F = 48.0, P = 0.0408;  t = 0.1429, df = 4, 95% CI = [-0.06145, 0.06812]  12hr: F = 3.250, P = 0.4706;  t = 2.910, df = 4, 95% CI = [0.001841, 0.07816]  24hr: F = Infinity, P < 0.0001;  t = 3.363, df = 4, 95% CI = [0.01220, 0.1278]  36hr: F = 3.880, P = 0.4098;  t = 2.626, df = 4, 95% CI = [-0.005556, 0.1989]  **MCM2**  8hr: F = 1.469, P = 0.8352;  t = 0.2219, df = 4, 95% CI = [-0.2686, 0.3153]  12hr: F = 1.865, P = 0.6980;  t = 2.089, df = 4, 95% CI = [-0.05594, 0.3959]  24hr: F = 5.571, P = 0.3043;  t = 14.30, df = 4, 95% CI = [0.2606, 0.3861]  36hr: F = 19.69, P = 0.0966;  t = 2.081, df = 4, 95% CI = [-0.09126, 0.6379]  **PCNA**  8hr: F = 109.8, P = 0.0181;  t = 0, df = 4, 95% CI = [-0.1948, 0.1948]  12hr: F = 1.0, P = 0;  t = 4.315, df = 4, 95% CI = [0.02614, 0.1205]  24hr: F = 6.143, P = 0.2800;  t = 2.121, df = 4, 95% CI = [-0.01544, 0.1154]  36hr: F = 1.346, P = 0.8526;  t = 5.369, df = 4, 95% CI = [0.1191, 0.3742]  **cycA2**  8hr: F = 1.147, P = 0.9316;  t = 0.01641, df = 4, 95% CI = [-0.5605, 0.5672]  12hr: F = 32.33, P = 0.06;  t = 0.4158, df = 4, 95% CI = [-0.2815, 0.2082]  24hr: F = 10.11, P = 0.1800;  t = 2.200, df = 4, 95% CI = [-0.01921, 0.1659]  36hr: F = 4.00, P = 0.40;  t = 7.099, df = 4, 95% CI = [0.1705, 0.3895]  **Ki67**  8hr: F = 5.073, P = 0.3293;  t = 1.224, df = 4, 95% CI = [-0.7407, 0.2874]  12hr: F = 1.6, P = 0.7691;  t = 0.3525, df = 4, 95% CI = [-0.2980, 0.3846]  24hr: F = 21.0, P = 0.0909;  t = 1.492, df = 4, 95% CI = [-0.02008, 0.06674]  36hr: F = 3.083, P = 0.4898;  t = 6.714, df = 4, 95% CI = [0.09188, 0.2215] |
| Fig. S6c | Two-tailed unpaired Student’s *t*-test | CDK4/6-independent: P = 0.0004  CDK4/6-dependent: P = 0.0184 | CDK4/6-independent: F = 148.5, P = 0.0134;  t = 11.10, df = 4, 95% CI = [29.70, 49.51]  CDK4/6-dependent: F = 1.677, P = 0.747  t = 3.842, df = 4, 95% CI = [3.235, 20.10] |
| Fig. S6d | Two-tailed unpaired Student’s *t*-test | Control: P = 0.6399  DHFR-p27: P = 0.0013 | Control: F = 5.377, P = 0.3136;  t = 0.5053, df = 4, 95% CI = [-3.626, 5.239]  DHFR-p27: F = 25.56, P = 0.0753;  t = 8.016, df = 4, 95% CI = [-27.09, -13.15] |
| Fig. S6e | Two-tailed unpaired Student’s *t*-test | Control: P = 0.8294  DHFR-p27: P = 0.0044 | Control: F = 1.372, P = 0.8432;  t = 0.23, df = 4, 95% CI = [-3.529, 2.989]  DHFR-p27: F = 16.88, P = 0.1119;  t = 5.79, df = 4, 95% CI = [-11.80, -4.150] |
| Fig. S7c | One-way ANOVA with Tukey’s post hoc analysis | Control-CDK4/6i+: P < 0.0001 Control-CDK4/6i+MEKi: P < 0.0001 CDK4/6i-CDK4/6i+MEKi: P = 0.0007 | Control-CDK4/6i+: 95% CI = [59.59, 80.76] Control-CDK4/6i+MEKi: 95% CI = [85.51, 106.7] CDK4/6i-CDK4/6i+MEKi: 95% CI = [15.34, 36.50] |
| Fig. S7e | Two-tailed unpaired Student’s *t*-test | **c-Myc**  DMSO vs Palbo: P = 0.9997  DMSO vs Palbo + Tram: P = 0.0060  Palbo vs Palbo + Tram: P = 0  .0059  **EU**  DMSO vs Palbo: P = 0.8831  DMSO vs Palbo + Tram: P = 0.0019  Palbo vs Palbo + Tram: P = 0  .0028 | **c-Myc**  DMSO vs Palbo: 95% CI = [-0.9269, 0.9136]  DMSO vs Palbo + Tram: 95% CI = [0.5731, 2.414]  Palbo vs Palbo + Tram: 95% CI = [0.5798, 2.420]  **EU**  DMSO vs Palbo: 95% CI = [-0.7909, 1.084]  DMSO vs Palbo + Tram: 95% CI = [0.9791, 2.854]  Palbo vs Palbo + Tram: 95% CI = [0.8325, 2.708] |

**Supplementary Data 1: Additional Statistical Information**.
