# Supplementary material for: Non-canonical pathway for Rb inactivation and external signaling coordinate cell-cycle entry without CDK4/6 activity
Source: Nat Commun. 2023 Nov 29;14:7847. doi: 10.1038/s41467-023-43716-y (PMC10687137; doi:10.1038/s41467-023-43716-y)
Supplement: Supplementary file 5 — Reporting Summary [file 41467_2023_43716_MOESM5_ESM.pdf]

nature portfolio

Corresponding author(s): Hee Won Yang

Last updated by author(s): Nov 6, 2023

Reporting Summary

Nature Portfolio wishes to improve the reproducibility of the work that we publish. This form provides structure for consistency and transparency in reporting. For further information on Nature Portfolio policies, see our Editorial Policies and the Editorial Policy Checklist.

Statistics

For all statistical analyses, confirm that the following items are present in the figure legend, table legend, main text, or Methods section.

n/a	Confirmed	
☐	☒	The exact sample size (*n*) for each experimental group/condition, given as a discrete number and unit of measurement
☒	☐	A statement on whether measurements were taken from distinct samples or whether the same sample was measured repeatedly
☐	☒	The statistical test(s) used AND whether they are one- or two-sided<br>*Only common tests should be described solely by name; describe more complex techniques in the Methods section.*
☒	☐	A description of all covariates tested
☒	☐	A description of any assumptions or corrections, such as tests of normality and adjustment for multiple comparisons
☐	☒	A full description of the statistical parameters including central tendency (e.g. means) or other basic estimates (e.g. regression coefficient) AND variation (e.g. standard deviation) or associated estimates of uncertainty (e.g. confidence intervals)
☐	☒	For null hypothesis testing, the test statistic (e.g. F, t, r) with confidence intervals, effect sizes, degrees of freedom and P value noted<br>*Give P values as exact values whenever suitable.*
☒	☐	For Bayesian analysis, information on the choice of priors and Markov chain Monte Carlo settings
☒	☐	For hierarchical and complex designs, identification of the appropriate level for tests and full reporting of outcomes
☒	☐	Estimates of effect sizes (e.g. Cohen's *d*, Pearson's *r*), indicating how they were calculated

Our web collection on statistics for biologists contains articles on many of the points above.

Software and code

Policy information about availability of computer code

Data collection	Quantitative real-time polymerase chain reaction was performed on a QuantStudio 6 Flex real-time PCR system (Applied Biosystems, #4485691). Fluorescence fixed-cell images were captured using a Nikon microscope (Nikon Instruments), mounted onto an inverted Eclipse Ti-2 body (Nikon) using 20× objective (Nikon CFI Plan Apo Lambda, 0.75 NA, 2-by-2-pixel binning). Fluorescence live-cell images were captured with a Nikon microscope using either a 20× or 10× (Nikon CFI Plan Apo Lambda, 0.45 NA, no binning) objective. Immunoblots were scanned with either an Odyssey Infrared Imaging System (LI-COR) for fluorescence-based detection or a ChemiDoc system (Bio-Rad) for chemiluminescence.
Data analysis	Quantitative real-time polymerase chain reaction was analyzed in Excel 2019 (Microsoft). Fluorescence images were analyzed using custom MATLAB scripts in MATLAB R2021a (MathWorks), followed by the analysis pipeline as described in Kim et al., 2023 (https://doi.org/10.1016/j.celrep.2023.113198). Imaging analysis code is available at https://github.com/tjdtn5160/image-analysis-kim-2023. Analysis of immunoblots was conducted using the Image Studio Lite software (LI-COR, version 3.1) for fluorescence-based detection or the Image Lab software (Bio-Rad, version 5.2.1) for chemiluminescence.

For manuscripts utilizing custom algorithms or software that are central to the research but not yet described in published literature, software must be made available to editors and reviewers. We strongly encourage code deposition in a community repository (e.g. GitHub). See the Nature Portfolio guidelines for submitting code & software for further information.

Data

Policy information about availability of data

All manuscripts must include a data availability statement. This statement should provide the following information, where applicable:

- Accession codes, unique identifiers, or web links for publicly available datasets
- A description of any restrictions on data availability
- For clinical datasets or third party data, please ensure that the statement adheres to our policy

The processed image data plotted in each figure and uncropped immunoblots are available in the Source Data file. Fluorescent blots are converted to gray scale. No original code is reported in this paper. The raw image data are available under restricted access due to large file size. Access can be obtained from the corresponding author upon reasonable request (Hee Won Yang: hy2602@cumc.columbia.edu).

Research involving human participants, their data, or biological material

Policy information about studies with human participants or human data. See also policy information about sex, gender (identity/presentation), and sexual orientation and race, ethnicity and racism.

Reporting on sex and gender	Not applicable to this study
Reporting on race, ethnicity, or other socially relevant groupings	Not applicable to this study
Population characteristics	Not applicable to this study
Recruitment	Not applicable to this study
Ethics oversight	Not applicable to this study

Note that full information on the approval of the study protocol must also be provided in the manuscript.

Field-specific reporting

Please select the one below that is the best fit for your research. If you are not sure, read the appropriate sections before making your selection.

☒ Life sciences ☐ Behavioural & social sciences ☐ Ecological, evolutionary & environmental sciences

For a reference copy of the document with all sections, see nature.com/documents/nr-reporting-summary-flat.pdf

Life sciences study design

All studies must disclose on these points even when the disclosure is negative.

Sample size	Sample size was determined by the maximum number of cells that our automated microscope could image in each experiment. In live-cell imaging, we captured images from 3 sites per well across 96 wells. For fixed-cell imaging, we captured from 32 sites per well, also across 96 wells.
Data exclusions	Data generated from unhealthy cells or failed experiments due to obvious mistakes such as equipment failure, cell contamination, misused reagent, and etc were excluded. Control conditions were used to determine whether cells were healthy or not.
Replication	All experiments were performed in biological replicates and such replicates were successful and reproducible. At least two independent experiments were conducted for each experiment. At least three independent experiments were performed to calculate significance. The number of replicates for each experiment can be found in the figure legends.
Randomization	The distribution of all samples into experimental groups was done randomly. For single-cell experiments, the seeding into 96-well plates was also randomized, and the selection of wells for treatment was performed in a random manner.
Blinding	Investigators were not blinded to cell lines receiving different treatments. Data reported are not subjective but rather based on quantitative measurement and automatic analyses.

Reporting for specific materials, systems and methods

We require information from authors about some types of materials, experimental systems and methods used in many studies. Here, indicate whether each material, system or method listed is relevant to your study. If you are not sure if a list item applies to your research, read the appropriate section before selecting a response.

Materials & experimental systems

n/a	Involved in the study
☐	☒ Antibodies
☐	☒ Eukaryotic cell lines
☒	☐ Palaeontology and archaeology
☒	☐ Animals and other organisms
☒	☐ Clinical data
☒	☐ Dual use research of concern
☒	☐ Plants

Methods

n/a	Involved in the study
☒	☐ ChIP-seq
☒	☐ Flow cytometry
☒	☐ MRI-based neuroimaging

Antibodies

Antibodies used

phospho-Rb (Ser807/811) (Cell Signaling Technology, #8516, 1:2000(IF)), Rb (Cell Signaling Technology, #9309, 1:2000(WB,IF)), c-Myc (Cell Signaling Technology, #5605, 1:1000(IF)), phospho-ERK1/2 (Thr202/Tyr204) (Cell Signaling Technology, #4370, 1:3000(WB),1:500(IF)), ERK1/2 (Cell Signaling Technology, #4696, 1:3000(WB), 1:500(IF)), phospho-AKT (Ser473) (Cell Signaling Technology, #4060, 1:2000(WB), 1:200(IF)), AKT (Cell Signaling Technology, #2920, 1:2000(WB)), PPARγ (Cell Signaling Technology, #2435, 1:1000(WB), 1:100(IF)), p21 (Cell Signaling Technology, #2947, 1:2000(WB)), p27 (Cell Signaling Technology, #3686, 1:2000(WB), 1:1600(IF)), p57 (Cell Signaling Technology, #2557, 1:250(IF)), GAPDH (Cell Signaling Technology, #2118, 1:5000(WB)), cyclin D1 (Thermo Scientific, #MA5-14512, 1:500(WB)), FPR1 (BioLegend, #391602, 1:1000(WB)), SYN1 (ABclonal, #A17362, 1:1000(WB)), Rb (Abcam, #ab181616, 1:2000(WB) in mouse and rat cell lines), c-Myc (Abcam, #ab32072, 1:1000(WB)), p21 (BD Biosciences, #556430, 1:500(WB)), p27 (BD Biosciences, #610241 1:300 (WB) in OP-9 cells), p21 (Santa Cruz Biotechnology, #sc-271610, 1:300(WB) in PC-12 and OP-9 cells), Alexa Fluor 488 goat anti-rabbit (Thermo Scientific, #A32731, 1:2000), Alexa Fluor 568 goat anti-rabbit (Thermo Scientific, #A11036, 1:2000), Alexa Fluor 488 goat anti-mouse (Thermo Scientific, #A32723, 1:2000), Alexa Fluor 568 goat anti-mouse secondary antibodies (Thermo Scientific, #A11031, 1:2000), Anti-rabbit HRP-linked antibody (Cell Signaling Technology, #7074, 1:2000), IRDye 800CW goat anti-mouse (LI-COR Biosciences, #926-32210, 1:2000), and IRDye 680RD goat anti-rabbit (LI-COR Biosciences, #926-68071, 1:2000).

Validation

All antibodies utilized are commercially available and have undergone validation by the manufacturers and/or our team for their intended applications within this study.
1. Phospho-Rb (Ser807/811) Rabbit mAb (#8516): Detects phosphorylated Rb at Ser807/811 without cross-reacting with Ser608 phosphorylation. Reactivity: Human, Mouse, Rat, Monkey.
2. Rb Mouse mAb (#9309): Binds total Rb protein, no cross-reactivity with p107/p130 homologues. Reactivity: Human, Monkey, Bovine, Pig.
3. c-Myc Rabbit mAb (#5606): Recognizes total c-Myc protein, not suitable for Myc-tagged proteins. Reactivity: Human, Mouse, Rat.
4. Phospho-ERK1/2 (Thr202/204) Rabbit mAb (#4370): Targets phosphorylated ERK1/2 without cross-reacting with other phosphorylated residues, verified by ERK inhibitors. Reactivity: Wide range including Human, Mouse, Rat, Hamster, and others.
5. ERK1/2 Mouse mAb (#4370): Detects total ERK1/2 protein, may preferentially bind p42/ERK2 over p44/ERK1. Reactivity: Human, Mouse, Rat, and others.
6. Phospho-AKT (Ser473) Rabbit mAb (#4060): Recognizes AKT1 phosphorylated at Ser473, also binds AKT2/AKT3, verified by AKT inhibitors. Reactivity: Wide range including Human, Mouse, Rat, and others.
7. Akt Mouse mAb (#2920): Binds total Akt protein, specific without cross-reactivity. Reactivity: Human, Mouse, Rat, Monkey.
8. PPARγ Rabbit mAb (#2435): Detects total PPARγ protein. Reactivity: Human, Mouse.
9. p21 Rabbit mAb (#2947): Recognizes total p21 protein, no cross-reactivity with other CDK inhibitors. Reactivity: Human, Monkey.
10. p27 Rabbit mAb (#3686): Detects total p27 protein, verified by Knockout. Reactivity: Human, Rat, Monkey.
11. p57 Rabbit pAb (#2557): Binds total p57 protein, does not recognize p27, verified by Knockout. Reactivity: Human.
12. GAPDH Rabbit mAb (#2118): Targets total GAPDH protein. Reactivity: Human, Mouse, Rat, and others.
13. Cyclin D1 Rabbit mAb (#MA5-14512): Specific to Cyclin D1/Bcl-1, verified by Knockdown. Reactivity: Human, Mouse.
14. FPR1 Mouse mAb (#391602): Quality control tested via immunofluorescent staining. Reactivity: Human.
15. SYN1 Rabbit pAb (#A17362): Targets SYN1, validated by western blot and ELISA. Reactivity: Mouse, Rat.
16. Rb Rabbit mAb (#ab181616): Specific to Rb, verified by Knockout. Reactivity: Human, Mouse, African green monkey.
17. c-Myc Rabbit mAb (#ab32072): Targets c-Myc, verified by Knockout. Reactivity: Human, Mouse, Rat.
18. p21 Mouse mAb (#556430): Specific to p21, verified by Knockout. Reactivity: Human, Mouse, Rat, and others.
19. p27 Mouse mAb (#610241): Targets p27, verified by Knockout. Reactivity: Human, Mouse, Rat, and others.
20. p21 Mouse mAb (#sc-271610): Recognizes p21, reactive across multiple species, verified by Knockout. Reactivity: Human, Mouse, Rat, and others.

Eukaryotic cell lines

Policy information about cell lines and Sex and Gender in Research

Cell line source(s)

1. ATCC: MCF-10A (#CRL-10317, human female), RPE1 (#CRL-4000, human female), HS68 (#CRL-1635, human male), H358 cells (#CRL-5807, human male), H1373 (#CRL-5866, human male), MP41 (#CRL-3297, human female), MP46 (#CRL-3298, human male), PC-12 (#CRL-1721, rat male), OP-9 (#CRL-2749, mouse embryo).
2. Rockland Immunochemicals: WM989 (#WM989-01-0001, human female), WM983B (#WM983B-01-0001, human male).
3. Dr. Galit Lahav's laboratory: MCF-7 (human female, doi.org/10.1038/ng1293)
4. Dr. Orion Weiner's laboratory: PLB-985 (human female, doi.org/10.1038/10042)

Authentication

We did not conduct further authentication for cells obtained from ATCC and Rockland. However, it's worth noting that both ATCC and Rockland authenticate their cell lines through STR analysis, as indicated on their product specification webpages.

The PLB-985 cell line has been identified as a misidentified subclone of the HL-60 cell lines in the ICLAC database. We've addressed this in the 'Cell culture' section of the methods.

Mycoplasma contamination	All cell lines tested negative for mycoplasma, as determined by PCR amplification of mycoplasma DNA.
Commonly misidentified lines (See ICLAC register)	The PLB-985 cell line has been identified as a misidentified subclone of the HL-60 cell line in the ICLAC database. PLB-985 cells are recognized models for human neutrophils. Their use was crucial to demonstrate in vitro cell differentiation across three distinct cell lines.
